# Data-Driven Structural Health Monitoring and Damage Detection through Deep Learning: State-of-the-Art Review

**DOI:** 10.3390/s20102778

**Published:** 2020-05-13

**Authors:** Mohsen Azimi, Armin Dadras Eslamlou, Gokhan Pekcan

**Affiliations:** 1 Department of Civil and Environmental Engineering, University of Nevada, Reno, NV 89557, USA; 2 Department of Civil Engineering, University of British Columbia, Vancouver, BC V6T 1Z4, Canada; 3 Department of Civil Engineering, Iran University of Science and Technology, Tehran 13114-16846, Iran; armin_dadras@civileng.iust.ac.ir

**Keywords:** deep learning, machine learning, structural health monitoring, crack detection, damage detection, data science, computer vision

## Abstract

Data-driven methods in structural health monitoring (SHM) is gaining popularity due to recent technological advancements in sensors, as well as high-speed internet and cloud-based computation. Since the introduction of deep learning (DL) in civil engineering, particularly in SHM, this emerging and promising tool has attracted significant attention among researchers. The main goal of this paper is to review the latest publications in SHM using emerging DL-based methods and provide readers with an overall understanding of various SHM applications. After a brief introduction, an overview of various DL methods (e.g., deep neural networks, transfer learning, etc.) is presented. The procedure and application of vibration-based, vision-based monitoring, along with some of the recent technologies used for SHM, such as sensors, unmanned aerial vehicles (UAVs), etc. are discussed. The review concludes with prospects and potential limitations of DL-based methods in SHM applications.

## 1. Introduction

Civil infrastructures are prone to a significant loss of functionality due to structural deficiencies that are primarily caused by material deterioration and loadings from earthquake, wind, vehicle, or ambient vibrations. In the United States, on a grade scale of A (excellent condition) to F (unacceptable condition), the overall score was as low as D+ for infrastructures, and C+ for bridges with an estimated $123Bn for retrofitting [1]. The report states that 7.5% of bridges rated structurally deficient and mostly below standard, with many elements approaching their end of service life. Furthermore, more than 30% of the approximately 617,000 highway bridges in the US need immediate attention due to deteriorating conditions [2].

During recent decades, ensuring life safety and the need to reduce inspection costs have emerged as the top priorities for practicing engineers and researchers. Therefore, the significance of cost-effective structural health monitoring (SHM) to ensure long-term structural integrity and safety levels has been highlighted on many platforms [3,4,5,6,7]. Various types of emerging SHM methods have the potential to streamline periodic inspections and minimize the direct and indirect costs that are associated with undesired failure of aging infrastructure in addition to conventional inspection and non-destructive evaluation (utilizing impact echo, ultrasonic surface wave, ground-penetrating radar, electrical resistivity, etc.) techniques [8]. At the center of any SHM method and application lies sensors and sensor data (observable response). Recent advancements in sensor and communication technologies (contact and contactless, wired and wireless, etc.) have created opportunities for the acquisition of observables at an unprecedented rate and amount. Furthermore, advancements in other supporting hardware and software have been exploited in various forms. For example, a major focus has been on the development of new technologies to efficiently maintain the infrastructures while using potentially viable alternatives, such as unmanned aerial systems (UAS). Current UAS research focuses on fully autonomous multirotor platforms that can hover for extended periods and carry a wide variety of sensors to enable SHM tasks. Such sensors may include high-resolution cameras for visible-spectrum light, as well as infrared and near-infrared cameras, LIDAR systems, radar, and sonar. A team of drones could inspect a bridge for defects with minimal or no management from humans on the ground [9].

The limitations on sensor measurement capabilities and challenges in deploying sensor networks due to power and data communication requirements have historically hindered the deployment of dense sensor arrays on civil infrastructure. Large amounts of heterogeneous data are becoming available from different types of sensors, as these limitations have been overcome. However, conventional SHM methods that rely on validated multi-physics models may generally not be suitable for effective health monitoring utilizing large sensor datasets. Fortunately, advances in data-driven techniques revolutionized data collection and interpretation. Unlike the traditional physics-based SHM models, data-driven models offer bottom-up solutions that include diagnosis and prognosis that include damage detection and remaining life estimation, respectively [10]. In addition, the traditional physics-based models require the least noise in the measured data, which is not possible when considering the nature of structures and working conditions. Consequently, data-driven models have demonstrated versatility and become the most attractive approaches in SHM [10,11].

Deep Learning (DL) is considered as a sub-branch of machine learning (ML), and its applications in dealing with large amounts of data have been successfully demonstrated on many platforms. The DL models can capture and learn information that is hidden in the data to predict different patterns via stacked blocks of layers that form the DL skeleton [10,12]. Fortunately, the advances in parallel computation, along with the development of DL [13], DL-based models have been successfully used in several applications in a wide range of research areas, including computer vision, speech, and audio recognition, and SHM. The main reasons for such significant attention toward DL-based SHM can be summarized, as follows:

**Advances in Big Data and cloud-based computation**: the cost of sensors has decreased significantly, while the cost of materials has been increasing in recent decades, which makes it possible to deploy large numbers of sensors in host structures and transfer data wirelessly to cloud-based fast computers. Similarly, the cost of portable devices and cameras has reduced, which makes it feasible to access and monitor parts of large-scale infrastructures through autonomous systems [14].

**Advances in computer hardware and software**: multi-core processors have dramatically improved during the last decade and, accordingly, there has been significant attention towards the exploitation of the capabilities of graphics processor unit (GPU) for training deep neural networks with minimum time while using dedicated software packages implemented in NVIDIA, Python, MATLAB, as well as the online cloud-based platforms, including Amazon and Google.

**Advances in Data Science**: data is considered as the core component of any SHM application [15]. The term ‘Data Science’ and ‘Data Engineering’ did not exist until a few decades ago, and now it is at the forefront of data-driven applications in several fields, including SHM. With the recent advances in ML algorithms as well as data acquisition and data transmission, data scientists can interpret data, detect abnormalities, or recover lost-data [16].

**Advances in Transfer Learning (TL):** while the DL-based research and applications are effectively becoming more widespread, the pre-trained networks, such as VGG [17], AlexNet [18], and ResNet [19], have been receiving increased attention for SHM-related applications, which has been frequently proven to be very effective and time-saving.

This paper provides an overview of frontier DL-based studies that made significant contributions to SHM until recently. The main goal of this review is to present the relative findings of the latest studies in SHM and assist researchers in this field with a condensed source of references that are related to novel DL-based SHM methods. The peer-reviewed papers were selected from prominent databases, including Science Direct, Web of Science, ASCE, Engineering Village, Sage, and Wiley Online Library. In Section 2, the review begins with the introduction of DL and outlining the main concepts. Section 3 and Section 4 review the recent application of DL techniques to vibration- and vision-based SHM, respectively. Applications of unmanned aerial vehicles (UAVs) and Smartphones as the new generation of devices that facilitate data acquisition, monitoring, and identification of damages are explained in Section 5. In Section 6, the Transfer Learning and the popular pre-trained networks in SHM are described. In Section 7, the explanations regarding data science as a necessary tool for data-driven SHM are presented and a comprehensive list of recently utilized datasets is reported. In Section 8, popular software applications for DL-based SHM is reviewed. Finally, Section 9 is provided to list the prospects as the big picture and highlights the main points of the paper.

## 2. From Shallow to Deep Learning: An Overview

Machine learning (ML) is a subcategory of artificial intelligence (AI). The goal of ML techniques is to develop trainable algorithms that are capable of learning from available measured or simulated response data, so that future predictions can be made [20,21]. The scope of ML methods is generally much broader in computer science than other engineering fields; however, only relevant aspects in SHM applications are discussed in this review. ML-based SHM models are mainly designed with the ability to learn by themselves and, from this point of view, DL can be considered as a feature-learning tool and a subset of ML [10,22].

ML-based SHM models can be categorized as supervised, unsupervised, or reinforcement learning. In supervised learning, an ML model can be trained whlie using a set of training data with labeled target values. If the outputs of the model are discrete or categorical variables, the model is known as a classifier (e.g., support vector machine, k-nearest neighbor, Bayesian method, decision trees, as well as deep learning); otherwise, the model performs regression (e.g., neural networks, decision trees, linear and nonlinear regression) [20,21]. The methods that are classified as unsupervised learning, cluster datasets without explicit training procedures. Hierarchical clustering, partitional clustering, k-means, spectral clustering, etc. are examples of clustering methods [22]. On the other hand, in reinforcement learning, the ideal performance is determined through agents, by try and error, which leads to maximizing the so-called reward. The performance limitations for traditional ML methods, such as designing and pre-selection of appropriate features, can be eliminated through DL. It is noted that the use of conventional models requires knowledge and experience for designing features for a specific SHM applications, which is not always practical as the monitored systems become more complex (e.g., due to highly nonlinear behavior), the implementation of conventional models requires knowledge and experience for designing features, which is not always practical [10].

On the other hand, the DL-based SHM methods aim to develop fully automated feature extraction and hierarchical representation mechanisms from the raw input data through stacked blocks of deep neural network (DNN) layers with nonlinear mappings [23,24]. Each layer in a DNN is responsible for learning a new representation of the input response data and, thus, DL-based SHM is an end-to-end system with no requirement for human intervention for designing features. Therefore, all of the feature extraction, pattern classification, and regression parameters are simultaneously designed [10], which makes DL-based SHM applicable to a wide range of problems with minimum knowledge about the specific features.

**Figure 1:**
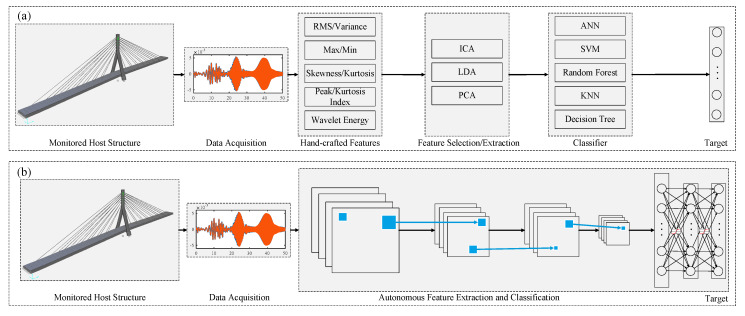
(**a**) Conventional data-driven SHM vs. (**b**) Deep learning-based SHM.

Figure 1 depicts the aforementioned contrast between conventional and DL-based SHM. In conventional data-driven SHM techniques: (1) expert knowledge is required to design the features; (2) a step-by-step training procedure is required; and, (3) trained models are generally less efficient for large-scale structures and may not be suitable for vision-based SHM applications. On the other hand, for the DL-based SHM: (1) training procedure has an end-to-end structure and features are automatically extracted; (2) hyper-parameters are trained simultaneously; and, (3) trained models are suitable for large-scale structures and efficient for vision- and vibration-based SHM while dealing with compressed or big data.

**Figure 2:**
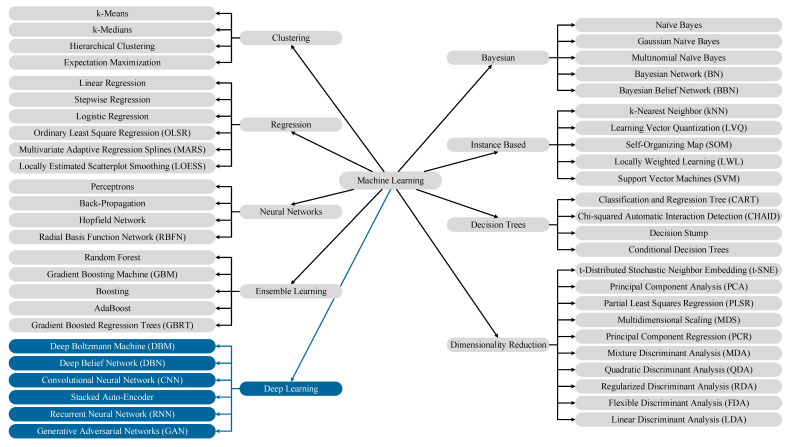
Machine Learning (ML) algorithms mind map.

Various alternative DL models have been recently proposed, such as Deep Convolutional Neural Networks [25], Deep Boltzmann Machines [26], Deep Belief Network [10], Recurrent Neural Networks [27], Auto-encoders [28], and Generative Adversarial Networks (GANs) [29], etc. The number of new ML algorithms has been increasing; however, a mind map of the frequently used algorithms, including deep learning (shown in dark shaded color), are presented in Figure 2.

Inspired by the significant advances in computer vision, researchers have recently attempted to solve civil engineering problems by adapting the vision-based deep learning methods. DL-based SHM techniques have been used for: general SHM [30], multi-level damage detection, corrosion detection [31], concrete surface bughole recognition [32], concrete crack detection [33], pavement crack detection [34], acoustic emissions source detection [35], etc. One common objective of these proposed approaches is to avoid traditional visual inspections by providing modern, economic, safe, fast, and autonomous methods that are suitable for any type and scale of structures [3,36,37]. Several disadvantages have been reported for direct methods in image-processing for crack detection. The main problem with the majority of the algorithms in the available literature is that the models are custom-made for certain datasets, which may have lower performance in real-world applications due to challenging circumstances that involve weather, temperature, camera position and quality, shadow and light, etc. [38]. In addition, such approaches highly depend on the selected pre-processing methods, including edge detection.

The following sections present the state-of-the-art applications of DL in SHM to provide a baseline for future studies. Investigations with similar goals and tools are compared in terms of model architecture, datasets, as well as their performance.

## 3. Vibration-Based SHM through DL

Numerous vibration-based damage assessment methods have been developed with particular applications in SHM while using ML techniques. DL has introduced new horizons in vibration-based data-driven SHM for large-scale structures and facilitated the acquisition and processing of large sets of data from different types of sensors [3,4,39]. Most of the conventional methods to localize damage, such as radiography or ultrasonic methods, require prior knowledge of the approximate location of damage [36,40,41]. Identifying candidate damage locations can be time-consuming, costly, and difficult. However, the vibration-based methods are founded on the premise that damages (physical changes) cause corresponding changes in vibration characteristics (especially modal shapes, frequencies, and damping) [40], and they may be used to identify the location of damage from measured response data. Kong et al. provide a detailed review of these techniques [42].

In general, vibration-based methods can be classified into two broad categories, namely model-based (parametric) and non-model-based (non-parametric). Model-based techniques usually require computational models and associated assumptions about the structural system as one key element. They usually yield good accuracy, but, in real-world applications, the essential and accurate information about the structural system might not necessarily be available. The difficulties in developing reliable computational models lead to the next category, so-called, non-parametric methods. These methods essentially perform post-processing of response (sensor) data to identify damages without any prior assumption regarding the structural system.

ML techniques contributed to both of these categories. They are usually used to extract the modal parameters in the scope of non-model-based methods [43,44]. The traditional ML methods involve two phases in non-model-based methods. The first phase is feature extraction, in which sensor data (e.g., acceleration) are used to extract effective features, thereby eliminating the cumbersome manual feature extraction process. The second phase is a classification procedure that identifies the location and/or level of damage [45]. Support Vector Machines (SVM), Probabilistic ANNs (PNN) [46,47,48], Fuzzy ANNs (FNN) [49], and Extreme Learning Machine Networks (e.g., online sequential) [50] are some of the popular methods that are used for vibration-based SHM.

**Figure 3:**
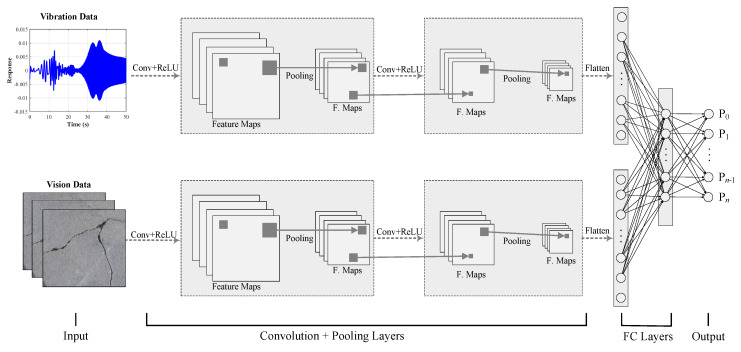
A multi-headed deep neural network for different input data.

Abdeljaber et al. [51] presented an approach for damage identification while using output-only response data. Training data were generated for various simulated damage (loose bolt) cases from measured acceleration response. After training separate CNNs for each damage case, the probability of damage (PoD) indicator was defined. Examining undamaged, single damage, and multiple damage cases, it was shown that specific cases were accurately identified with a 0.54% average error. In a similar study, three-dimensional wireless sensors were used to record the acceleration response [52]. However, this approach required a large amount of response data that were associated with various permutations of loose connections that rendered the application impractical. Abdeljaber et al. [53] introduced a new approach that only required two states of damage, namely undamaged and fully-damaged cases, in order to alleviate the drawbacks. The computational procedure was similar to the previous investigation, but this method could only determine the general condition of the structure. Lin et al. [54] trained a CNN simulating FE model of a simply supported beam and considering noisy and noise-free states. It was demonstrated that the response frequency bands, vibration modes, and their combination were learned by a deep neural network as essential characteristics that were identifiable from sensor data. Wang and Cha [55] proposed an unsupervised method using an acceleration signal that was obtained from an intact laboratory-scale three-dimensional (3D) steel bridge. The response signal vectors were normalized, and then the continuous wavelet transformation (CWT) and Fast Fourier Transformation (FFT) were applied, which were then fed to a two-dimensional (2D)-CNN autoencoder to extract essential features. Ten One-Class Support Vector Machines (OC-SVM) were used as novelty-detectors corresponding to the sensors. The location of sensors with the highest novelty rates was considered to be the approximate location of loose-bolt damage. Wavelet packet transform (WPT) of vibration signals, as well as vision data, are efficient in damage localization, according to the studies that were conducted by Pan et al. [20,21] and Pan and Yang [56]. A parallel configuration of CNN (Figure 3) can be used for a robust damage localization and damage intensity estimation, where the time-domain data are processed using the one-dimensional (1D) CNN (upper branch) and the time-frequency-domain (WPT) or vision data are processed using 2D CNN (lower branch), and the feature maps are concatenated in the end for classification or regression. Details of the CNN configuration in Figure 3 are available from Azimi and Pekcan [57].

Bao et al. [30] proposed a framework for anomaly detectiosn that is inspired by human vision and thinking. First, the measured acceleration response data were transformed to grayscale and then fed into deep convolution neural networks (DCNNs) after manual labeling. The method reached a global accuracy of 87.0% for one-year data testing that can be used for real-time SHM, alarming systems, and off-line assessment. Tang et al. [58] enhanced this method by working with different forms of response data. First, two images of time and frequency domains were stacked with, respectively, red and green channels into dual-channel images. In contrary to the previous work using imbalanced data (i.e., with the unequal number of samples for different classes), herein data were balanced and its impact was investigated. The method achieved 93.5% accuracy and outperformed the former approach. Wu and Jahanshahi [59] presented a study on the application of CNN for linear and nonlinear structural dynamic response estimation and identification. They examined single- and multi-degree of freedom systems and indicated that, in some cases, trained convolution kernels and convolution layers can be interpreted as numerical integration or dominant frequency extraction operators, respectively. Furthermore, they compared the results that were obtained by the MLP technique with CNN and showed that the latter approach is more accurate and robust against noise-contaminated input data. Oh et al. [60] proposed a CNN model to estimate the response of tall buildings under wind excitations. They showed that integration can be approximated by convolutional layers without a max-pooling layer. They provided a physical interpretation of the trained convolutional layers indicating their ability of noise filtering, eliminating irrelevant information, and preserving the dominant frequency. Recently, Khodabandehlou et al. [61] applied 2D CNN for the overall assessment of concrete bridges while using shake-table tests of a one-fourth scale highway bridge. They implemented a 2D CNN for damage classification that was trained using 40 sets of experimental acceleration records and tested on eight new sets. Four system-level damage states namely intact, minor, moderate, and extensive were quantified and accurately predicted by the proposed model. Li and Sun [62] applied CNN to the damage detection of an experimental cable bridge model, which compared the performance of CNN with those of random forest, support vector machine, k-nearest neighbor, and decision tree methods showing at least 15% outperformance in the accuracy.

In general, these methods demonstrated that CNNs require a large amount of data for training. These necessary data can be generated from finite element (FE) simulations, or from the measured response (acquired via sensors when available). In FE simulations, an exhausting amount of response data associated with different damage states can be easily generated, which yields accurate and high-level damage identification subject to the accuracy of the simulation models. In other words, it should be taken into consideration that these data differs from the recorded sensor data because of the uncertainties and noise. The recorded response data are usually utilized for level 1 (presence of damage) identification through an unsupervised scheme. Pathirage et al. [63] introduced an approach to remove these drawbacks based on autoencoders. The first-order sensitivity-based method was used for matching the FE model and the real model [64]. Subsequently, the calibrated FE model was utilized to extract frequencies and mode shapes as training data. They proposed a two-step framework comprising dimension reduction and relationship learning DNNs. In the first step, a deep autoencoder was used to extract salient features and its outputs fed into a damage identification network. A pre-training scheme was used to find the optimal weights. They considered the uncertainty effect in the FE model as well as noise. It was shown that the proposed approach was more accurate than the traditional ANN. Pathirage et al. [28] added a pre-processing stage and introduced a three-step method with data pre-processing, sparse dimensionality reduction, and relationship learning steps. This framework was similar to the former method and it demonstrated efficient and acceptable performance. Recently, Teng et al. [65] similarly utilized the simulation data of modal strain for training a CNN and verified its performance while using experimental response data from a steel frame. They achieved 100% accuracy in damage localization of several single- and multi- damage scenarios. In most of the studies, the geometric location of sensors was not considered in the input data structure. Providing a solution to feed this information is of sufficient importance to influence accuracy. For this purpose, Sajedi and Liang [66] developed a grid environment methodology for the real-time damage segmentation in large scale civil infrastructures. They used a fully convolutional encoder–decoder neural network that was trained by cumulative intensity measures as the input and damage states of nodes as output. Their proposed approach yielded global accuracies of 96.3% and 93.2% for the detection of damage location and severity in a FE model, respectively.

Several researchers attempted to employ other types of sensor data or use alternative features since the acceleration response signal is highly prone to noise. Li et al. [67] collected deflection data of a scaled-down model bridge through a fiber-optic gyroscope. They fed these data as input to a 1D-CNN to classify its damage as four classes comprising an intact class and other three damaged states. They examined the performance by cross-validation and demonstrated that the CNN had at least 15.3% accuracy advantage over other traditional techniques, such as random forest, support vector machine (SVM), k-nearest neighbor (KNN), and decision trees (DT). Lopez-Pacheco et al. [68] proposed a new frequency domain convolutional neural network (FDCNN) for damage identification based on the Bouc–Wen hysteretic model to increase robustness against noise. The FDCNN utilized spectral pooling operator, attenuated the noise in measurements, and was trained four times faster than similar time-domain networks. Moreover, it was demonstrated that energy dissipation could be captured by FDCNN, which allowed for higher diagnosis accuracy.

Hung et al. [69] developed a hybrid framework combining 1D-CNN and Long-Short Term Memory (LSTM) network for damage detection. This network directly receives raw time-series data and determines the presence of damage. It was shown that, with a low level of noise, the proposed network could provide accurate detections. Ding et al. [70] created a sparse Deep Belief Network (DBN) based on Restricted Boltzmann Machines (RBM) and trained by incomplete modal data that were extracted from FE simulations. The introduced network could successfully predict the damage location and severity with acceptable accuracy, even in multi-damage cases and in the presence of noise. Although their method showed better performance than swarm intelligence techniques, it is noted that the latter techniques require fewer finite element simulations and they are able to adapt to different types of structural systems. Therefore, instead of directly identifying the damage attributions, DNN can be used for other purposes, such as denoising, to enhance the performance of other available techniques. Fan et al. [71] introduced a modified version of Residual Convolutional Neural Network (ResNet) with dropout, skip connection, and sub-pixel shuffling modules to denoise acceleration response signals. They tested the trained network on extensively contaminated data that were measured from a TV Tower and observed that the suggested approach could successfully identify the modal properties of the structure.

## 4. Vision-Based SHM through DL

The majority of the conventional infrastructure inspection techniques are based on visual assessments (i.e., crack existence, location, and width) that rely on experts’ insight and experience, which may not always be reliable [72]. Besides, such techniques are costly and time-consuming [73]. The vision-based inspections can be performed by inspecting raw images (by human and without post-processing), by image enhancement or applying basic image processing filters to magnify and detect edges to accelerate the inspection, and by autonomous image processing tools that require computers and ML algorithms [74,75].

The main goal of the studies in the computer vision field concerned with information extraction from image data to automatically recognize the real-world (as visual cortex functions). Such efforts were initiated decades ago with the aim of detection of edges, and it has been continuously developed to problems with complex image patterns, such as facial recognition, vehicle, and pedestrian detection [39]. Jahanshahi and Masri [76] showed that morphological operations are not the only techniques in image processing-based damage detection. Other approaches include: binarization [77], image correlation [78,79], edge detectors [36,80,81,82], percolation model [83], fractal analysis [84], etc.

Most of the efforts in computer vision are concentrated in developing end-to-end learning algorithmswhile using artificial intelligence, particularly through DCNNs that was capable of achieving more than 95% of accuracy on image-based classification problems [85]. In addition, the application of CNNs has been extended to pixel-level labeling within an image to detect and localize different objects of interest while using nonlinear filters and feature maps [27]. The recent advances in computer-vision have brought more attention to such technologies in SHM as one of the most effective tools in the non-contact assessment of deflection [86,87], corrosion [88], concrete spalling [89,90], concrete and pavement cracks [33,91,92], fatigue detection [93], and surface and subsurface damages [94,95]. Dorafshan et al. carried out a comprehensive study [96] to discuss the performance of vision-based image processing techniques in SHM, which employ artificial intelligence.

### 4.1. Crack Detection through Vision-Based DL

Infrastructures, particularly aging concrete structures, are prone to the formation of cracks due to changing loading conditions, corrosion, etc. Cracks in concrete or road pavements usually appear as lines with random orientations and intensity. Usually, these lines are darker and connected, and a simple crack detection can be carried out while using properly prescribed thresholds. Generally, two approaches have been used by researchers in vision-based SHM, particularly in crack detection, the image binarization method [97], and the sequential image processing method [83]. Binarization techniques for transforming images into black and white pixels, or cracked and sound pixels, [98], as well as mathematical morphology [99] can facilitate and improve the accuracy of the detection process due to the nature of cracks.

**Figure 4:**
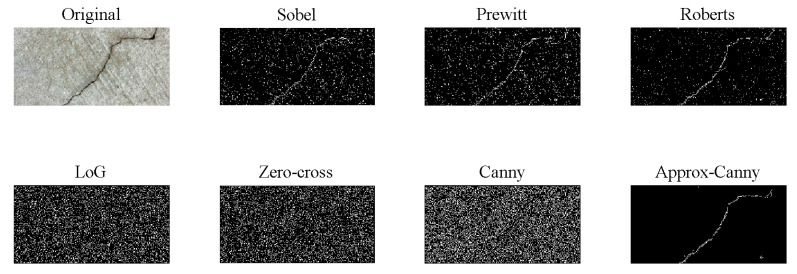
Concrete surface crack detection using different edge detection filters.

Prior to the introduction of DL in crack detection, the traditional approaches have been using pixel groups with similar color levels. The earlier generation of heuristic methods for vision-based crack detection in concrete structures was based on edge detection algorithms by applying filters, such as Roberts, Prewitt, Sobel, and LoG (in the spatial domain), or Butterworth and Gaussian (in the frequency domain) [100]. Figure 4 compares the results of applying different filters for detecting cracks on a concrete surface.

**Figure 5:**
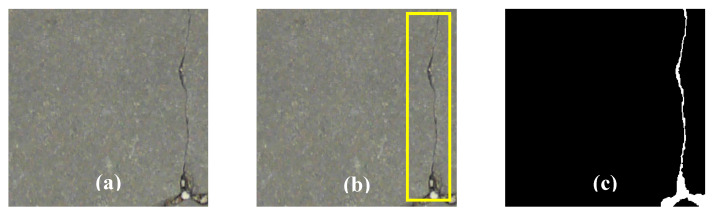
Illustration of (**a**) crack and non-crack image classification, (**b**) object localization, and (**c**) pixel-level segmentation (open-access images from Yang et al. [101]).

Abdel-Qader et al. [80] showed that the fast Haar transform has higher accuracy (86%) as compared to the other filters, such as Canny and Sobel, with 76% and 68% of accuracies, respectively. The image dataset that was used in this study, as well as the classification criteria, were further improved by Dorafshan et al. [96]. In general, major ML-based problems include three techniques: classification, localization, and segmentation. Figure 5 illustrates the frequent crack detection approaches: classification [25], object localization [23], and pixel-level segmentation [101]. Using the classification method, the dataset is labeled as cracked, non-cracked (sound). In the crack localization method, the cracks within each input image are labeled with bounding boxes. In the pixel-level segmentation method, the pixels are classified as cracked and non-cracked [102].

Dorafshan et al. discussed a comparison of different edge detection methods and performance of different filters have been discussed by [96]. Based on ANN-based image processing methods, several researchers highlighted potential applications of autonomous crack detection techniques. Jahanshahi and Masri [103] proposed ML-based models using SVMs for concrete crack detection, based on morphological features. The crack width was measured by identifying the centerline of cracks in their study. Using the abovementioned techniques, an automated vision-based crack detections framework was proposed by Yeum and Dyke [104] for bridge inspection.

Only a limited number of studies have attempted to compare the performance of recently developed crack detection methods by other researchers [96,97,105]. In addition, most of the recent studies have not clearly described the accuracy and classification criteria, including true positive (TP) metrics for reproducibility of the results. Furthermore, a comparison of several studies from a broadly different range of datasets [106], as well as comparisons using small datasets or the idealized datasets that were collected in laboratory conditions [96] do not reflect the merits of one method over another. Dorafshan et al. [37] and Talab et al. [83] proposed an automatic crack detection using the OTSU threshold [107] and image filtering. Such methods were later improved by implementing terrestrial laser scanning, which has three main steps: shading correction, crack detection, and mapping [108], and could be implemented in an automated manner using robotic systems yielding up to 95% accuracy [109].

Deep CNNs have been consistently developed by researchers in the computer vision field; Rawat and Wang present more details regarding the background of the CNN developments in image classification [110]. Following such significant achievements, several studies adapted CNN to detect surface and subsurface cracks in pavement and concrete. Earlier studies used DCNN to classify concrete or pavement surfaces by sliding-window method, but, recently, semantic segmentation through an end-to-end pipeline using fully convolutional networks (FCN) has attracted attention [111,112]. This approach has been employed to tackle challenging classification problems in different fields, including SHM. Recently, Chen and Jahanshahi [113] developed an enhanced CNN-based crack detection method while using a Naïve Bayes data fusion scheme for the extracted data from video frames. Kim et al. [73] proposed a faster CNN-based model to determine pixel-wise location while using image binarization.

Detecting cracks in tunnels is of vital importance. They might be a potential sign of a hidden danger that can pose serious threats to users or even become a trigger to the catastrophic collapse. On the other hand, identifying tunnel cracks is challenging, because there are many noise patterns in the tunnel images. Therefore, developing automated accurate methods for monitoring their surfaces can effectively enhance safety and decrease the potential costs. Li et al. [114] created a database of 60,000 tunnel crack images for training, testing, and comparing different crack segmentation networks. According to their study, by introducing clique block and attention mechanisms into U-net, it can significantly outperform basic U-net, fully convolutional networks (FCN), SegNet, and multi-scale fusion crack detection (MFCD) for detecting cracks in tunnel noisy images.

Soloviev et al. [115], Li et al. [116], Tong et al. [117], and Fan et al. [118] demonstrated the use of DCNNs to detect and recognize cracks as defects with quantifiable properties in applications for crack detection on pavement surfaces (e.g., crack length and size). Fan et al. [119] proposed a CNN-based multi-label classifier by improving the positive-to-negative ratio of samples. In another study by Wang et al. [120], they proposed a CNN model with three blocks of convolutional layers followed by two FC layers, consisting of 1,246,240 trainable parameters in total, which could detect surface cracks from the subdivided images of asphalt pavement. Tong et al. [117] developed another two-stage CNN-based model to also detect asphalt pavement crack length. A fast pavement crack detection network (FPCNet) was developed by Liu et al. [121] using encoder-decoder configurations.

The majority of studies in the literature validated the performance of DL models implemented in laboratory conditions with image datasets of intact and cracked surfaces, which still has limitations in addressing the real-world conditions. The acquired surface images may be contaminated with noise, shadow, dust, or extra brightness, which requires more robust and intelligent techniques for classification. Depending on the applications, such practical challenges have been addressed in several studies. Kim and Cho [122] defined a five-class crack detection problem using a large volume of images collected from the Internet as well as their augmentations. Their study considered field conditions to tackle real-world limitations that are associated with several uncertainty factors, as well as the inability in employing contextual information, such as the nature of materials, structural components, and the region of interest (ROI). Cha et al. discussed the feasibility of autonomous DL-based methods for crack detection, and Cha and Choi [123] proposed CNN-based classifiers and applied a sliding window method on 256 × 256 RGB images of concrete surfaces to detect cracks. Their proposed methods achieved an accuracy of 97% for concrete image datasets when considering different light intensities associated with variable weather conditions. Jang et al. developed a DL-based crack detection method [124] using hybrid images of combined vision and infrared thermography of macro- and micro-cracks. They observed that the hybrid images made the network robust against varying operational conditions such as shadow, dust on the surface, rust, etc. Moreover, they developed a sticking-type UAV that can be utilized in the inspection of large reinforced concrete civil infrastructures. Jang et al. [125] devised a ring-type robot for crack evaluation of circular bridge piers in a controlled manner. This robot provides fast scanning and high-quality raw images for crack detection. Feeding these images, they trained a CNN that was able to precisely segment the crack maps on the piers. The proposed system could identify images with 97.47% recall and 90.92% precision, according to the experimental results.

In typical region-based classification, or object detection, a bounding box is created around the region of interest (e.g., cracks, spalling, components, etc.) [96]. For example, Ali et al. [126] proposed a modified cascade face detection method that uses the Viola–Jones algorithm for crack detection on concrete walls while using bounding boxes around the region of the crack. This method was modified by Ramana et al. [127,128] to automatically detect loosened bolts in steel structures with higher efficiency when compared to the earlier studies using hand-crafted features [129].

Yeum et al. used region-based CNNs (R-CNN) [130] for post-event evaluation of buildings with an accuracy of nearly 60%; however, this technique requires further developments to also include multiple damage scenarios. Xu et al. proposed a fast R-CNN approach [131] to detect different damage types in concrete structures as well as damage locations using bounding boxes. Fast R-CNN and Faster R-CNN were developed by Girshick [132] and Ren et al. [133], respectively. Another newly-developed region-based segmentation technique is Mask R-CNN [134] that segments images into objects, which can be used for crack detection, concrete spalling, and rebar detection. Cha et al. proposed a faster R-CNN for detecting multiple damage types, and Cha et al. developed the method to localize multiple damage types, including steel and bolt corrosion and delamination. One of the main drawbacks of the regular CNN approaches for detecting cracks is their deficiency in specifying out-of-plane cracks. Deng et al. [135] recently embedded deformable modules into various R-CNN and fully convolutional networks to overcome this drawback. When comparing the suggested technique with regular networks, they observed that the modified approach not only improves the detection accuracy of out-of-plane cracks, but also enhances the accuracy for other cases.

Other studies proposed pixel-level classification methods [33] to provide more precise information regarding the path and intensity of cracks. In most of the published research, the binary classification problems include distinguishing ‘crack’ and ‘non-crack’ regions or pixels. For more precise classification, Dung and Anh [33] proposed semantic segmentation to also identify path and density,. The typical object detection models attempt to fit a bounding box around the ROI [85], and semantic segmentation methods [136] or pixel-level classification [101], should be used to precisely delineate damage level, shape, and location. For pavement crack detection problems, Zhang et al. [27,137] proposed CrackNet, an efficient model based on R-CNNs. Xu et al. [93] developed a DL-based fatigue crack identification technique for long-span steel box girder bridges using deep CNN, as well as a framework for steel crack detection while using restricted Boltzmann machine [138] with high accuracy. Hoskere et al. proposed a pixel-wise DCNN with a parallel configuration and a fully CNN (FCN) [139,140] to localize and classify different damages, including concrete cracks, spalling, exposed rebars, corrosion, fatigues cracks, and asphalt cracks.

### 4.2. Structural-Component Recognition and Change Detection through Vision-Based DL

It is essential to perform a global-level inspection, as well as the structural component recognition process, before moving closer to the details, to understand the relationship between damage and safety of structures [86]. However, recent studies in DL-based SHM have not fully addressed this concern. Even the video-based crack detection models do not interpret the impact of damage in a global context. Yeum et al. [141] proposed a CNN-based technique to classify civil infrastructure images by recognizing regions of interest. Gao and Mosalam used similar object-detection techniques [142] to classify structural components as well as damage types. A Faster R-CNN algorithm was used by Liang [143] to automatically detect structural components of the RC bridge system using boundary boxes.

**Figure 6:**
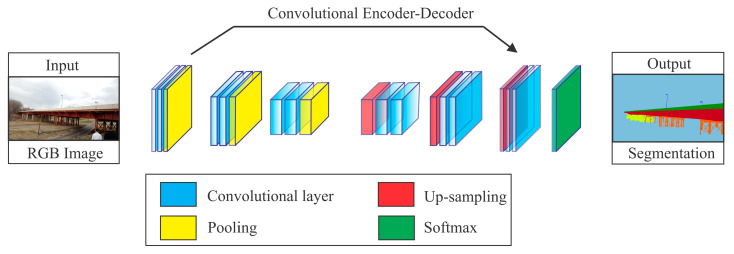
Illustrations of bridge components recognition.

Narazakia et al. [144] used both global and close-up views to train two recurrent neural networks (RNNs) while using a single image-based pre-trained FCN for structural component recognition from video image data. The simple RNN and ConvLSTM units in their models could learn memories of the focus region of the video. The ground-truth labels for the video frames of flying UAV were synthetically created using a game engine [85]. Their overall goals were associated with the recognition, localization, and structural component classification from complex scenes. Each input image is automatically or semi-automatically down-sampled using convolutional layers and then up-sampled to generate the segmented image that is similar to the ground truth, as shown in Figure 6.

Alcantarilla et al. proposed street-view (ground-level) change detection [145] using deconvolutional networks. Using a CNN model, Stent et al. [146] proposed a change detection method for tunnels. The main assumptions in these studies were that the cracks are connected slender and darker lines on concrete surfaces [83].

## 5. Applications of UAVs and Portable Smartphones for DL-Based SHM

Deep learning networks facilitated the damage identification task by automating the process and achieving acceptable levels of accuracy. However, in some cases, inspectors do not have access to all parts of structures to acquire image data (for vision-based approaches) or sensors data (for vibration-based methods). This is one of the main difficulties in structures, such as tall buildings, bridges, and heritage structures [147]. Drones were proposed as tools for inspecting such structures to overcome these difficulties [148]. Drones, Unmanned Aerial Vehicles (UAVs), or Unmanned Aerial Systems (UASs), are classified based on their level of automaticity, size, and other capabilities. They minimize the need for physical labor in addition to being time-saving, cost-effective, safe, available, and accurate. In recent years, different studies have been conducted in order to provide a framework for using UAVs, showing their applicability and address some of their disadvantages. When considering the rapid developments in drone industries, nowadays, utilizing intelligent UAVs for wireless data acquisition is not considered as a future technology.

The main developers of UAVs for bridge and other structural inspections are the departments of transportation (DOT) and the universities in the USA [85,149,150,151,152]. Along with the developments in wireless data transmission techniques, several studies have been conducted that utilized UASs technologies to broaden vision-based inspection in SHM [14,153,154], as well as vibration-based techniques [155].

Since DL-based SHM is an emerging technology, the capability of UAVs for robust real-time evaluation is still a challenge. Kim et al. [122] demonstrated the feasibility of UAV-based inspection of a concrete retaining wall. They analyzed videos (two frames/second) that were recorded from approximately 2m from the concrete surface. By embedding image processing and computer vision systems in UAVs, instant crack detection tasks could be safely done with minimum costs and maximum greater accuracy [156]. For example, Jang et al. [157] mounted a hybrid image scanning system (HIS) combining the laser thermography cameras and vision sensors in order to detect concrete cracks.

For bridge inspections, Kim et al. [158] deployed a high-resolution camera on a commercial UAV to collect images for crack detection and generating damage map of a concrete bridge. Kang and Cha [14,159] developed an autonomous UAV system for SHM while using ultrasonic beacons to replace the role of GPS that performs poorly in partially covered places, such as under bridge decks. In addition, Huynh et al. [160] used a UAV for the quasi-real-time inspection of connection bolts on a full-scale girder bridge. Dorafshan et al. [75] examined the performance of different UASs for detecting cracks in steel bridges and concluded that instability in GPS-denied and windy environment might pose major challenges for UAS-assisted inspections.

UAVs were also employed to create image datasets for masonry heritage structures [161]. The images collected by UAVs are sometimes noisy or have relatively low contrast; in addition, the unavailability of GPS signals in indoor environments or under bridges interrupts their performance [162]. Duarte et al. [163] discussed the performance of the networks when considering multi-resolution images derived from satellite, manned, and unmanned aerial vehicles. Hoskerre et al. [140] proposed a framework to convert UAV data to DL-based condition-aware models for automating and accelerating post-earthquake inspections. They trained three networks for building information, the presence of damage, and the types of damage. Moreover, they suggested an approach for modal identification of the structures while using videos recorded from different parts of a structure through a divide and conquer strategy [164]. Because of the growing attention to smartphone applications, several studies used them as inexpensive tools for SHM [102]. Images taken by smartphones have been utilized for the identification of various damages, such as pavements [165], bolt loosening [160,166], volumetric damages [167], and concrete cracks. Zhao et al. [168] developed a mobile-based method for measuring the forces in cables of cable-stayed bridges.

Using the framework of Core ML as well as the Xcode, Li, and Zhao [169,170] integrated a trained CNN model into a developed smartphone application in order to detect the presence of concrete surface cracks on a bridge with 99% accuracy. Furthermore, using CNN, Wang et al. [171] developed a real-time efflorescence and spalling detection of historic brick masonry buildings. Based on 99 × 99 RGB images that were acquired by low-cost smartphone sensors, Zhang et al. [34] developed a deep CNN model with six convolution layers for automatic crack detection on road surfaces, which was a binary classification task. Pauly et al. proposed a deeper CNN model [172] to enhance the performance of CNNs based on the 99 × 99 RGB images. Maeda et al. [173] prepared a large-scale dataset while using a smartphone that was installed on a car dashboard, which used images to develop an end-to-end public application so-called ‘RoadDamageDetector’, which classifies different types of road damages.

## 6. Transfer Learning (TL) through Pre-Trained Models

**Figure 7:**
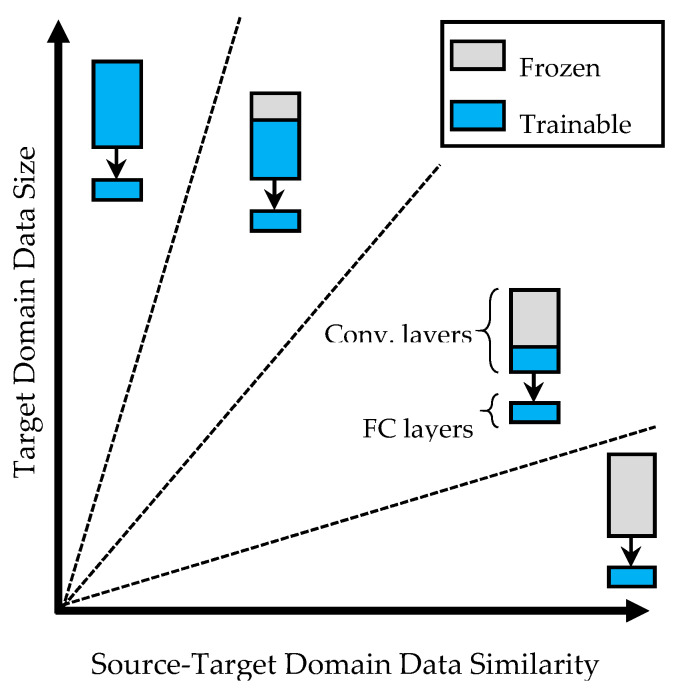
Four transfer learning approaches based on target domain size and similarity to the source domain [57].

When the dataset is relatively small and there is a pre-trained network that has already been trained on a larger dataset, an efficient way is to fine-tune the existing network for the new similar classification task. Using transfer learning techniques (TL), the training time can be minimized by transferring the coefficients from the base model instead of starting with randomly assigned weights. The depth of the network has a direct relationship with the number of training parameters. Therefore, deep networks require a considerably long time and a large amount of data for training. Transfer Learning (TL) can alleviate this issue by providing prior knowledge (weights) that was obtained from a similar problem [174]; therefore, fine-tuning can be carried out with a lower computational cost and fewer data samples. Figure 7 illustrates four typical TL strategies thatare based on the target data size in its similarity to the source domain. For a CNN model with convolutional layers in series, followed by fully connected (FC) layers, a common practice is to fine-tune the last FC layers, or replacing them with new layers. Therefore, the convolutional layers are frozen and skipped during the training.

The application of TL is an emerging area in SHM, and novel studies are being carried out in classification problems in vision-based SHM [142]. Pre-trained CNN models can even be used for new problems with completely different output classes. In pavement crack detection, TL has been proven to be an efficient approach for improving the accuracy of the classification problem [175]. For example, Gopalakrishan et al. [176] were able to perform crack detection on Hot-Mix Asphalt, as well as Portland Cement Concrete, through the TL technique. They used the VGG16 network that was trained on ImageNet data. Dorafshan et al. [96] compared the performance of a fully trained AlexNet model with the same AlexNet, but in transfer learning and no-training modes for concrete crack detection tasks. Perez et al. [177] applied a pre-trained VGG-16 for localization of building deteriorations that stem from dampness such as stain, peeling, and crazing. Özgenel and Sorguç [178] conducted a comprehensive study with an emphasis on the dataset size, number of training epochs, number of convolution layers, as well as the trainability and transferability features of each CNN-based pre-trained models. Wu et al. [179] designed an efficient DCNN that was developed using transfer learning (of VGG16 and ResNet18) and Taylor expansion-based network pruning. The network pruning technique refers to removing the least important neurons and filters of a belief network. They showed that the proposed approach reduces memory demands and inference time. This technique can be applied to decrease the need for a huge amount of training data without losing performance in damage detection. Table 1 provides further examples of TL applications using the popular pre-trained DNNs for SHM problems. The most popular pre-trained DNNs that have been frequently used for SHM problems are summarized in the following:

**AlexNet**: AlexNet [18], one of the earlier DL models, has been developed to classify objects in the images, and it won the ImageNet [180] classification competition in 2012. It has five convolutional and max-pooling layers, three fully-connected layers, and a 1000-way softmax output layer (25 layers in total). When considering the concrete surface cracks as objects, AlexNet can be fine-tuned for crack detection purposes through transfer learning [96,122]. AlexNet can be loaded to Matlab or Python using the dedicated toolboxes.

**VGG**: VGG16 [17] is a deeper version of AlexNet, which itself has six different configurations namely A, A-LRN, B, C, VGG16, and VGG19. VGG16 and VGG19 are the popular versions with 16 and 19 layers, and 138 and 144 million parameters, respectively.

**Inception**: the kernel size is related to the distribution of salient information. The large and small kernel sizes are suitable for global and local distribution of information, respectively. Using different filter sizes in parallel can resolve the problem of choosing suitable sizes. Different versions of inception modules, so-called V1, V2, V3, and V4, have evolved iteratively [181].

**ResNet**: Resnet50 [182] is a deep network that implements residual learning. It was introduced by Facebook AI Research (FAIR). Although it provides significantly high accuracy, it requires considerable processing time because of the significant depth of the network. ResNet50 has 50 main layers and 177 in total, and ResNet101 has 101 main layers with 347 layers in total [19].

**GoogleNet**: GoogleNet was proposed by Szegedy et al. [19]. It has 22 main layers (144 in total) [182] but 12 times fewer parameters than AlexNet. Using weighted Gabor filters [183] with various sizes in the inception sparse architecture allows for a deeper and wider network without increasing the computational budget.

**ZFNet**: ZFNet is the fine-tuned version of AlexNet, which was the Winner of ILSVLC 2013 in image classification. The Architecture of AlexNet and ZFNet is similar, and the differences are mainly focused on filter size and stride that resulted in reducing the error rates [184].

**CrackNet**: the first version of CrackNet (CrackNet I) was developed for crack detection on three-dimensional (3D) asphalt surfaces with the explicit objective of pixel-perfect accuracy [27,137]. This CNN does not use max-pooling layers and it achieved 90.13% precision and 87.63% recall. The second version (CrackNet II) enhanced the CrackNet I in terms of robustness against noise and increasing performance speed by removing the handcrafted feature generation, adding learnable parameters, and increasing the depth of the network. This CNN achieved 90.20% precision and 89.06% recall, which are better than the original version [185].

## 7. Databases for DL-Based SHM

Data are an essential part of all data-driven techniques in SHM. During recent years, the importance of reliable data for SHM applications has become obvious along with the emerging DL-based algorithms that can process and interpret massive amounts of data [15]. To date, no open-access and standard dataset for DL-based SHM has been published; however, individuals have created datasets generated under laboratory-controlled environments for limited applications. For example, Maguire et al. [206] created a database, called SDNET2018, which contains 56,000 images of a bridge deck, wall, and pavement, which is suitable for DL-based applications. The dataset by Hoskere et al. [139] consists of images from different types of structures, such as buildings, bridges, dams, pavements, as well as laboratory tests images. For small size datasets, the augmentation techniques can be applied, including geometry transformation, color conversion, and adding noise or blurring, which address the camera position and image quality as well as the light and weather conditions in real-world [122]. More sophisticated techniques, such as a generative adversarial network (GAN), have been used for data augmentation for structural images [207]. Table 2 and Table 3 summarize the vibration-based and vision-based datasets that have been recently used in DL-based SHM. The goal of the studies, as well as the nature of the datasets, size, and other characteristics, are also included.

## 8. Software Frameworks for DL Applications

Various frameworks have been developed by research centers to perform deep learning tasks. Table 4 summarizes the most popular ones (Pouyanfar et al. [220]) that continue to evolve.

**TensorFlow**: a powerful platform that is developed by Google and dedicated to deep learning applications is TensorFlow [221]. TensorFlow can deploy multiple CPUs or GPUs while using the same Application Programming Interface (API), which increased its popularity among the DL researchers [222]. No framework is superior over the others; however, some researchers compared the performances on a single GPU, and showed that the TensorFlow might not outperform Theano, Torch, Neon, and TensorFlow DL frameworks, despite its flexibility [105,223]. On the other hand, it is faster when Long Short Term Memory (LSTM) units are used as the core of the model [222]. It is worth noting that TensorFlow can also run models on mobile platforms.

**Torch/PyTorch**: Torch is a simple, open-source, and extensible DL framework that is dedicated to building fast and efficient GPU-based ML algorithms [222]. It outperforms CPU-based training according to Bahrampour et al. [105]. PyTorch is considered to be the primary software tool for deep learning after TensorFlow, which is developed by Facebook services.

**Keras**: one of Python’s most popular and high-level libraries in DL that is capable of running on top of TensorFlow [224]. Keras is extremely user-friendly and, due to the modularity and extensibility features, it is attractive for both novice and experienced researchers in DL.

**Caffe**: for computer-vision tasks, Caffe, which is developed at the University of California-Berkeley, is one of the fastest and easiest platforms to train CNNs with the capability of processing 60 million input images per day [225].

**Theano**: originally developed to be used as a CPU/GPU compiler in Python, and not to be a DL framework, but it is one of the Python libraries for fast numerical computations, particularly non-standard DL models [105]. For relatively shallow models of CNNs and LSTMs, Theano might outperform Torch and TensorFlow [105]. Theano is not further developed since 2017.

## 9. Summary and Prospects

The advantages of machine learning (ML) in solving structural health monitoring (SHM) problems are evident, and the number of deep learning (DL) applications has grown exponentially since its introduction during recent years. However, every proposed method has its limitations, and some of the major challenges are summarized, as follows:

1. Despite remarkable advances since the introduction of DL-based SHM, current techniques in the literature cannot be considered to be fully automated, and human perception is not easy to replicate through vibration or vision-based DL algorithms [226]. Such capabilities should be addressed in future studies, including the significance of damage with respect to the type of structural components, materials, locations, and other environmental conditions.
2. The number of available image databases of structural systems and other infrastructure components is very limited for SHM purposes, which leads to lower performance of the available trained models when new conditions arise, such as texture, joints, light, environment, pollutions, etc. In addition, environment-related issues cannot be perfectly simulated via generalized numerical models; therefore, larger datasets can only be formed by acquiring data from the real world.
3. The nature of damages, as well as their significance, may differ from one structural component to another when considering the global structural context. Therefore, comprehensive hierarchical approaches should be devised in which structural component recognition is included as an essential first step before using image data. Such an approach has the potential to bring context in the assessment, evaluation, and interpretation of damage as part of SHM applications.
4. Laboratory conditions are idealized in the available studies, and further research is needed for in-situ DL-based SHM. For example, the presence of wind and light may disrupt UAVs for the vision-based measurements. A clear example is the dynamic vibrations and deflections of a long suspension bridge under traffic and wind loads. In addition, damages occur gradually with small changes [145,146].

Along with the rise of the DL-based methods, the importance of data is more evident than ever before. Vision and vibration data from host structures both need to be automatically processed and stored efficiently, which requires robust and real-time signal and image processing models that are capable of identifying an anomaly in data. Response data compression and response reconstruction [227] for long-term monitoring purposes remain challenges that require more developments to avoid information loss. The near-future directions of DL-based technologies can be grouped into the following categories when considering the limitations and advantages of the recently proposed methods.

### 9.1. DL Applications in Vision-Based and Vibration-Based SHM

The DL-based damage detection techniques are capable of learning implicit relations between features with less computational costs. DL-based techniques result in significantly higher accuracy, despite the conventional ML methods that rely on hand-crafted features. Along with the advances in computer vision techniques, vision-based SHM is expected to play a pivotal role in the next generation of SHM. In addition, it has been shown that vibration data can be treated in grid-like images to train deep CNN models [61], which expands DL applications to solve real-world problems. In addition, DL models can be used to predict or minimize the response of complex structures without the need for complex finite element models.

### 9.2. DL Applications in SHM Using Self-Powered Sensors

During the last decade, wireless sensors networks (WSNs) have been developed as the best alternative for traditional wired networks. In addition, the development of self-powered sensors for harvesting energy from the sensed vibrations has recently been in the center of attention. Such technology has transformed data acquisition and promoted energy-efficient data interpretation [228]. The majority of the ML-based SHM methods are combinations of hand-crafted formulas and empirical models, which still depend on the knowledge of the inspector as well as the capability of computers and algorithms, despite the significant advances in data-driven approaches [22]. As the predominant methods in SHM, ML-based approaches require a large amount of data from the host structure. The incorporation of wireless networks of self-powered sensors with deep learning technologies will increase the efficiency and minimize the cost of maintenance through continuous monitoring. DL-based methods are reliable and efficient end-to-end training models with higher prediction accuracies, which are able to learn the nonlinear interrelation without the need for manual feature extraction methods.

### 9.3. DL-Based SHM as Part of IoT and Smart Cities

Emerging wireless data transmission and cloud-based computation have created new paradigms, known as the Internet of Things (IoT) [22]. Nowadays, it is practically feasible to mount low-cost wireless sensors in large numbers on infrastructures to efficiently monitor the structural health [229]. With the advantages of the powerful cloud-based computing and DL platforms, future studies would integrate DL and IoT for SHM to extract information from a large amount of data that are constantly received from networks of sensors. Such studies would further expand the boundaries of SHM and IoT in a large-scale, by remote sensing and monitoring, as well as learning from previous events to make decisions in the future. Smart and sustainable cities have been at the center of attention since the introduction of the IoT concept. Interpreting a large amount of distributed data requires efficient DL approaches that, ultimately, result in the integrity of the whole system with minimum costs and in an automatic way. The future studies would introduce new protocols in data acquisition, transmission, storing, and DL-based interpretation for SHM as part of IoT.

### 9.4. DL Applications in SHM through Transfer Learning/Synthetic Simulation Data

It is obvious that data play a key role in deep learning, and, as explained in previous sections, data collection is not always possible. One option is using transfer learning to shortcut the learning process by using some level of knowledge from earlier studies. An emerging technique for increasing the training data size is using synthetic data for pre-training DL models while using finite element analysis packages (for vibration-based data) or game engines (for vision-based data). Therefore, such data can be used for pre-training a network before fine-tuning using real-work data.

### 9.5. DL-Based SHM Using Portable Smartphones, UAVs and GNSS

In the current era, cell phones have become an integral part of our lives. These programmable small devices have a camera, memory, as well as good computing and network technology, which enables offline and cloud-based real-time assessments. The smartphones are generally equipped with different sensors, namely magnetometer, gyroscope, accelerometer, and GPS. These capabilities have made them a potential device for structural health monitoring, particularly, when they are paired with UAVs to obtain and process data while using DL-based algorithms. Moreover, the Global Navigation Satellite System (GNSS) technology that is capable of acquiring massive amounts of accurate structural vibration response data. These data can lead to noticeable gains in the operational efficiency of SHM. The cost of SHM can be reduced and its reliability can be improved, as this modern technology and DL will be linked together with a resulting decrease in the need for sensors [230].

### 9.6. DL-Based Seismic Vibration Control for Smart Structures

Time-delay and sensor failure are among the major issues that directly impact the performance of vibration control systems during an earthquake [231]. By implementing recurrent neural networks (RNNs), such as long short-term memory (LSTM) networks, it can be possible to predict the response of structures to address delay problems. Furthermore, signal failures or damage presence can be effectively detected, which leads to the design of stable and robust controllers [232]. Model-free deep reinforcement learning techniques for seismic vibration control are novel and they can incorporate deep learning models for training controllers by learning from experiments, which is suitable for active and semi-active control problems [233]. With the recently developed open-source Python packages, such as OpenseesPy [234] and OpenAI Gym [235], it is possible to build, train, and validate a deep reinforcement learning algorithm for nonlinear structures under seismic excitations.

## Figures and Tables

**Table 1:** Examples of Transfer Learning (TL) Applications in SHM using pre-trained deep neural networks (DNNs).

Pre-Trained Network	Purpose/Application	Researches in SHM
VGG (VGG-16, 19) [17]	Crack detection Mixed reality systems Bolt loosening detection Corrosion detection Component recognition Steel damage condition assessment Post-earthquake assessment	[32,34,102,141,144,145,176,178,186,187,188,189,190,191,192,193,194]
Inception (Inception-V2, V3, V4) ^1^ [19,181,195]	Crack detection Damage detection of historic masonry buildings	[33,169,176,196,197]
ResNet (ResNet-20, 50, 101, 152) [185]	Crack detection Bridge component extraction Structural inspection	[33,139,144,171,178,190,191,194,198,199,200]
AlexNet [18]	Crack detection Comprehensive maintenance and inspection sUAS-assisted structural inspections Post-earthquake assessment	[73,96,122,124,143,178,193,196,201,202,203]
GoogleNet [19]	Crack detention Post-disaster inspection Comprehensive maintenance and inspection for bridges	[143,157,169,178,196,198,202]
MobileNet	Road damage detection	[173]
UNet, SegCaps, SegNet [113]	Segmentation Pixel-level crack detection	[186,194]
ZF-net [187]	ZF-Net for fast R-CNN. Region-based DL for detecting multiple damage types Detection and localization of multiple types of damage Comprehensive maintenance and inspection for bridges Volumetric damage quantification	[167,203,204,205]
CrackNet, CrackNet-R [139,188]	Crack detection Pixel-level road crack detection	[27,101,194]

**^1^** V denotes the version.

**Table 2:** Examples of vibration-based datasets for deep learning (DL)-based SHM.

Reference(s)	Goal	Dataset
Zhang et al. [208]	Vibration-based structural state identification	8595, 14,465, and 4800 raw acceleration data (9 Ch. × 10,000) for each of the bridges
Pathirage et al. [28]	Damage identification by making a deep mapping between the modal characteristics and structural damage	20,000 data samples containing the first three frequencies and mode shapes obtained by Eigen analysis of finite element model
Avci et al. [52]	Wireless vibration-based bolt loosening detection	330 signals each containing 245,760 samples of velocity
Pathirage [63]	Vibration-based damage detection and finding the stiffness reduction of elements	Modal information of 10,300 damage cases that include the first seven frequencies (7 arrays) and the regarding mode shapes at 14 beam-column joints (98 arrays)
Tang et al. [58]	Data anomaly detection and classification	10,014 time and frequency response of a long-span cable-stayed bridge stacked in two channels with the resolution of 100 × 100
Wang and Cha [55]	Vibration-based loosened bolt localization	6800 frequency domain 50 × 50 matrices calculated by Fast Fourier Transformation (FFT) of acceleration signals of a lab-scale bridge
Yu et al. [209]	Damage identification and localization of buildings controlled with smart devices	1900 group of 5 × 2832 matrices of power spectral density
Lin and Nie [54]	Vibration-based feature extraction for damage detection	459 set of vertical acceleration signals collected from nine nodes in 1024 × 9 matrices
Bao et al. [30]	Vision-based anomaly detection and classification in a long-span cable-stayed bridge	333,792 of acceleration signals plotted in 100 × 100 one channel images
Abdeljaber et al. [53]	Bolt loosening localization on a lab-scale steel grandstand simulator	749 × 12 vectors of acceleration signals with 128 × 1 dimension

**Table 3:** Examples of vision-based datasets for DL-based SHM.

Reference(s)	Goal	Dataset
Gulgec et al. [210]	Robust damage detection and localization of steel connections	30,000 damaged and 30,000 healthy strain distribution matrices in 28 × 56 dimension
Ye et al. [211]	Concrete crack detection	14,000 concrete crack images with 80 × 80 pixel resolutions obtained from a concrete beam test
Xu et al. [131]	Multi-type seismic damage identification and localization	2400 images with 640 × 640 pixel resolution of concrete cracking, concrete spalling, rebar exposure, and rebar buckling
Nahata et al. [193]	Post-earthquake damage extent identification of buildings	1200 RGB images with 224 × 224 × 3 pixel resolution
Beckman et al. [167]	Concrete spalling damage detection and quantification	444 concrete spalling images with the resolution of 853 × 1440 pixels
Jang et al. [157]	Detection of micro and macro concrete cracks	20,000 hybrid combining vision and infrared thermography of concrete crack and intact images with 224 × 224 pixel resolution
Dung and Anh [33]	Concrete crack detection, segmentation, and density evaluation	A public dataset of 40,000 concrete crack 227 × 227 pixel images
Zhang et al. [166]	Real-time autonomous bolt loosening detection	300 tight and loosened bolt images with 224 × 224 pixels
Wang et al. [171,196]	Spalling detection for historic masonry structures	500 images with 500 × 500 pixel resolutions
A vision-based [189]	Crack detection of gusset plate welding in steel bridges	12,896 images with 64 × 64 pixels of cracks and the same number of non-cracks
Liu and Zhang [192]	Image-driven low cycle fatigue-induced damage identification for post-hazard inspection	8259 images with 224 × 224 pixels extracted from a the video was taken during the experimental test
Ni et al. [198]	Concrete thin crack identification and width measurement	65,319 crack and 64,681 non-crack 224 × 224 RGB images for GoogleNet and 60,000 images for ResNet
Hoskere et al. [140]	Rapid and autonomous post-earthquake inspections including identification of damage presence and damage type	A set of 665 images with 288 × 288 pixels containing post-earthquake damage scenarios such as concrete cracks, spalling and exposed rebar
Chen [113]	Inspection of nuclear power plants and detection of cracks in video frames	A total 147,344 crack and 149,460 non-crack 120 × 120 image patches
Liang [143]	Post-disaster system-level failure analysis of bridges, the structural component-level identification, and local-level damage localization	492 number of 224 × 224 RGB images
Zhao et al. [202]	Classification the types of bridges, recognition of bridge components and crack detection	3832 RGB images of an arch, suspension and cable-stayed bridges with 227 × 227 pixel resolution and 60,000 intact and cracked concrete RGB images with 224 × 224 pixel resolution
Gao et al. [142,212]	Component type, spalling condition, damage level, and damage type determination.	2000 images with a size of 224 × 224 RGB (Structural ImageNet)
Dorafshan et al. [203]	Autonomous inspection of concrete structures using Unmanned Aerial Systems (UASs)	9011 227 × 227 pixel images of lab-made bridge decks including 1471 cracked and 7540 intact cases taken by Nikon camera
Suh and Cha [204]	Damage type detection and localization	2366 images of concrete cracks, steel delimitation, corrosion, and bolt loosening with a size of 500 × 375 pixel
Wang et al. [196]	Damage type identification (intact, crack, efflorescence, and spall) and localization for masonry historic structures	5145 stretcher and header brick images with 480 × 105 and 210 × 105 pixel resolutions respectively
Kim et al. [72]	Determining the existence and location of cracks from surface images considering crack-like noise patterns	3186 images crack and intact surfaces with different distances between the camera and 227 × 227 pixel
Silva et al. [213]	Automated inspection of concrete structures and crack detection	3500 sample of concrete surface images of 256 × 256 pixels with and without cracks
Dorafshan et al. [96]	Image-based crack detection in concrete structures	18,000 concrete panel images with the size of 256 × 256 pixel simulating reinforced concrete bridge decks
Sharma et al. [214]	Image-based detection of the crack presence	15,600 crack and non-crack 28 × 28 RGB image patches
Li and Zhao [169]	Crack detection of concrete surfaces	real concrete surface RGB images with 224 × 224 pixel resolution and taken by a smartphone
Kang and Cha [14]	Autonomous UAV method using ultrasonic beacons for damage detection and localization	40,000 cracked and intact concrete surface images with 256 × 256 pixel
Yang et al. [101]	Semantically identification and pixel-wise segmentation	A collection of 800 images of various cracks with 224 × 224 pixel
Kim and Cho [122]	Crack detection on concrete surfaces	7195 images of cracks, joints, edges, plants, and intact surfaces with 227 × 227 pixel scraped from the Internet
Beckman et al. [205]	Volumetric damage detection and quantification	444 images of concrete spalling with the resolution of 853 × 1440 pixels
Kumar et al. [215]	Automated CCTV inspection of sewer pipelines	12,000 images of cracks, root intrusions, deposits, and in pipelines with the dimension of 256 × 256
Narazaki et al. [144,190,199]	Structural bridge components recognition	39,081 images of a concrete girder bridge with a size of 240 × 320
Karaaslan et al. [186]	Mixed reality inspection including detection and segmentation of cracks and spalls	51,300 concrete crack, road damage and bridge inspection images with the size of 300 × 300
Oliveira et al. [216]	Damage classification in an aluminum plate	720 frames of 128 × 128 greyscale representation of electromechanical impedance
Li et al. [217]	Concrete bridge inspection and estimating the probability of being cracked	326,000 samples of 18 × 18 one channel greyscale patches of concrete cracks and non-cracks
An et al. [124]	Autonomous detection of macro- and micro-cracks	A set of 20,000 images of crack and intact images 227 × 227 RGB images
Duarte et al. [163]	Building damage (rubble piles, debris) classification and assessment from images such as	A total of 12,973 of the satellite and airborne 224 × 224 pixel images
Ji et al. [218]	Identification of collapsed buildings from post-event satellite images	613 collapsed and 1857 non-collapsed buildings Post-Earthquake Satellite images with the resolution of 96 × 96
Kang and Cha [159]	Intact and cracked concrete area classification	A broad variation of 2304 × 1280 raw images of concrete surfaces
Modarres et al. [219]	Crack detection of concrete bridges and composite panels	2400 number of real concrete crack and intact surfaces and 6000 debonded and intact sandwich panels with a resolution of 96 × 96 pixel
Yeum et al. [141]	Classification and localization for visual assessment of connections in a full-scale truss structure	100,000 images of welded joints with a dimension of 256 × 256
Hoskere [139]	Damage localization and classification for post-earthquake structural assessment	1695 images of concrete spalling, exposed rebar, steel corrosion, concrete cracks, fatigue cracks and asphalt cracks with a resolution of 288 × 288
Nazaraki et al. [191]	Pixel-wise bridge component recognition	11,897 urban, bridge and general 180 × 180 images
Atha and Jahanshahi [31]	Assessment and corrosion detection on metallic surfaces	67,187 images of regions with and without corrosions with 128 × 128 pixel resolution
Cha et al. [25]	Automatic concrete crack detection	40,000 images of cracks on concrete images with a dimension of 256 × 256 pixel

**Table 4:** Comparison of popular deep learning frameworks.

Framework	Core Programming Language	Interface Support	CNN and RNN Support
TensorFlow	Python, C++, Cuda	Python, C/C++, Java	Yes
Torch/PyTorch	C, Lua	Python, C/C++, Lua	Yes
Keras	Python	Python, Matlab	Yes
Caffe	C++	Python, Matlab	Yes
Theano	Python	Python	Yes

## References

[1] ASCE ASCE’s 2017 Infrastructure Report Card. https://www.infrastructurereportcard.org/.

[2] FHWA Bridge Condition by Highway System 2019. https://www.fhwa.dot.gov/bridge/nbi/no10/condition19.cfm.

[3] Sohn H., Farrar C.R., Hemez F.M., Shunk D.D., Stinemates D.W., Nadler B.R., Czarnecki J.J. (2003). A review of structural health monitoring literature: 1996–2001. Los Alamos Natl. Lab..

[4] Salawu O. (1997). Detection of structural damage through changes in frequency: A review. Eng. Struct..

[5] An Y., Chatzi E., Sim S.H., Laflamme S., Blachowski B., Ou J. (2019). Recent progress and future trends on damage identification methods for bridge structures. Struct. Control Health Monit..

[6] Mashayekhi M., Santini-Bell E. (2019). Three-dimensional multiscale finite element models for in-service performance assessment of bridges. Comput.-Aided Civ. Infrastruct. Eng..

[7] Mashayekhizadeh M. (2018). Fatigue Assessment of Complex Structural Components of Steel Bridges Integrating Finite Element Models and Field-Collected Data. Bridge Struct..

[8] Cicero T., Cawley P., Simonetti F., Rokhlin S.I. (2009). Potential and Limitations of a Deconvolution Approach for Guided Wave Structural Health Monitoring. Struct. Health Monit..

[9] Rakha T., Gorodetsky A. (2018). Review of Unmanned Aerial System (UAS) applications in the built environment: Towards automated building inspection procedures using drones. Autom. Constr..

[10] Zhao R., Yan R., Chen Z., Mao K., Wang P., Gao R.X. (2019). Deep learning and its applications to machine health monitoring. Mech. Syst. Signal Process..

[11] Kaveh A., Dadras A. (2018). Structural damage identification using an enhanced thermal exchange optimization algorithm. Eng. Optim..

[12] Schmidhuber J. (2015). Deep learning in neural networks: An overview. Neural Netw..

[13] LeCun Y., Bengio Y., Hinton G. (2015). Deep learning. Nature.

[14] Kang D., Cha Y.J. (2018). Autonomous UAVs for Structural Health Monitoring Using Deep Learning and an Ultrasonic Beacon System with Geo-Tagging. Comput.-Aided Civil Infrastruct. Eng..

[15] Bao Y., Chen Z., Wei S., Xu Y., Tang Z., Li H. (2019). The State of the Art of Data Science and Engineering in Structural Health Monitoring. Engineering.

[16] Fan G., Li J., Hao H. (2019). Lost data recovery for structural health monitoring based on convolutional neural networks. Struct. Control. Health Monit..

[17] Very Deep Convolutional Networks for Large-Scale Image Recognition. https://arxiv.org/abs/1409.1556.

[18] Krizhevsky A., Sutskever I., Hinton G.E. ImageNet classification with deep convolutional neural networks. Proceedings of the Advances in Neural Information Processing Systems.

[19] Szegedy C., Liu W., Jia Y., Sermanet P., Reed S., Anguelov D., Erhan D., Vanhoucke V., Rabinovich A. Going deeper with convolutions. Proceedings of the IEEE Conference on Computer Vision and Pattern Recognition.

[20] Pan H., Azimi M., Gui G., Yan F., Lin Z. Vibration-Based Support Vector Machine for Structural Health Monitoring. Proceedings of the International Conference on Experimental Vibration Analysis for Civil Engineering Structures.

[21] Pan H., Azimi M., Yan F., Lin Z. (2018). Time-Frequency-Based Data-Driven Structural Diagnosis and Damage Detection for Cable-Stayed Bridges. J. Bridge Eng..

[22] Salehi H., Burgueno R. (2018). Emerging artificial intelligence methods in structural engineering. Eng. Struct..

[23] Cha Y.J., Choi W., Suh G., Mahmoudkhani S., Büyüköztürk O. (2018). Autonomous structural visual inspection using region-based deep learning for detecting multiple damage types. Comput.-Aided Civ. Infrastruct. Eng..

[24] Mosalam K., Muin S., Gao Y. (2019). New Direction in Structural Health Monitoring. NED Univ. J.Res..

[25] Rafiei M.H., Khushefati W.H., Demirboga R., Adeli H. (2017). Supervised Deep Restricted Boltzmann Machine for Estimation of Concrete. ACI Mater. J..

[26] Zhang A., Wang K.C., Fei Y., Liu Y., Chen C., Yang G., Li J.Q., Yang E., Qiu S. (2019). Automated pixel-level pavement crack detection on 3D asphalt surfaces with a recurrent neural network. Comput.-Aided Civ. Infrastruct. Eng..

[27] Pathirage C.S.N., Li J., Li L., Hao H., Liu W., Wang R. (2019). Development and application of a deep learning–based sparse autoencoder framework for structural damage identification. Struct. Health Monit..

[28] Goodfellow I., Pouget-Abadie J., Mirza M., Xu B., Warde-Farley D., Ozair S., Courville A., Bengio Y. Generative adversarial nets. Proceedings of the Advances in neural information processing systems.

[29] Bao Y., Tang Z., Li H., Zhang Y. (2019). Computer vision and deep learning–based data anomaly detection method for structural health monitoring. Struct. Health Monit..

[30] Atha D.J., Jahanshahi M.R. (2018). Evaluation of deep learning approaches based on convolutional neural networks for corrosion detection. Struct. Health Monit..

[31] Wei F., Yao G., Yang Y., Sun Y. (2019). Instance-level recognition and quantification for concrete surface bughole based on deep learning. Autom. Constr..

[32] Dung C.V., Anh L.D. (2019). Autonomous concrete crack detection using deep fully convolutional neural network. Autom. Constr..

[33] Zhang L., Yang F., Zhang Y.D., Zhu Y.J. Road crack detection using deep convolutional neural network. Proceedings of the 2016 IEEE International Conference on Image Processing (ICIP).

[34] Ebrahimkhanlou A., Salamone S. (2018). Single-Sensor Acoustic Emission Source Localization in Plate-Like Structures Using Deep Learning. Aerospacecraft.

[35] Dorafshan S., Maguire M., Qi X. (2016). Automatic surface crack detection in concrete structures using OTSU thresholding and morphological operations. UTC Rep..

[36] Kaneko S., Oka S., Matsumiya N. (2012). Detection of cracks in concrete structures from digital camera images. NTT Tech. Rev..

[37] Wu L., Mokhtari S., Nazef A., Nam B., Yun H.-B. (2014). Improvement of crack-detection accuracy using a novel crack defragmentation technique in image-based road assessment. J. Comput. Civ. Eng..

[38] Carden E.P., Fanning P. (2004). Vibration based condition monitoring: A review. Struct. Health Monit..

[39] Doebling S.W., Farrar C.R., Prime M.B. (1998). A summary review of vibration-based damage identification methods. Shock. Vib. Dig..

[40] Xu J., Fu Z., Han Q., Lacidogna G., Carpinteri A. (2018). Micro-cracking monitoring and fracture evaluation for crumb rubber concrete based on acoustic emission techniques. Struct. Health Monit..

[41] Kong X., Cai C.S., Hu J. (2017). The state-of-the-art on framework of vibration-based structural damage identification for decision making. Appl. Sci..

[42] Goh L., Bakhary N., Rahman A., Ahmad B. (2012). Prediction of unmeasured mode shape using artificial neural network for damage detection. J. Teknol..

[43] Hakim S., Razak H.A., Ravanfar S. (2015). Fault diagnosis on beam-like structures from modal parameters using artificial neural networks. Measurement.

[44] Castellini P., Revel G.M. (2000). An experimental technique for structural diagnostic based on laser vibrometry and neural networks. Shock. Vib..

[45] Jiang S.F., Zhang C.M., Koh C. (2006). Structural damage detection by integrating data fusion and probabilistic neural network. Adv. Struct. Eng..

[46] Jiang S.F., Zhang C.M., Yao J. (2011). Eigen-level data fusion model by integrating rough set and probabilistic neural network for structural damage detection. Adv. Struct. Eng..

[47] Zhou X., Ni Y., Zhang F. (2014). Damage localization of cable-supported bridges using modal frequency data and probabilistic neural network. Math. Probl. Eng..

[48] Palomino L.V., Steffen V., Finzi Neto R.M. (2014). Probabilistic neural network and fuzzy cluster analysis methods applied to impedance-based SHM for damage classification. Shock. Vib..

[49] Meruane V. (2015). Online sequential extreme learning machine for vibration-based damage assessment using transmissibility data. J. Comput. Civ. Eng..

[50] Abdeljaber O., Avci O., Kiranyaz S., Gabbouj M., Inman D.J. (2017). Real-time vibration-based structural damage detection using one-dimensional convolutional neural networks. J. Sound Vib..

[51] Avci O., Abdeljaber O., Kiranyaz S., Hussein M., Inman D.J. (2018). Wireless and real-time structural damage detection: A novel decentralized method for wireless sensor networks. J. Sound Vib..

[52] Abdeljaber O., Avci O., Kiranyaz M.S., Boashash B., Sodano H., Inman D.J. (2018). 1-D CNNs for structural damage detection: Verification on a structural health monitoring benchmark data. Neurocomputing.

[53] Lin Y.z., Nie Z.h., Ma H.w. (2017). Structural damage detection with automatic feature-extraction through deep learning. Comput.-Aided Civ. Infrastruct. Eng..

[54] Wang Z., Cha Y.j. Automated damage-sensitive feature extraction using unsupervised convolutional neural networks. Proceedings of the Sensors and Smart Structures Technologies for Civil, Mechanical, and Aerospace Systems.

[55] Pan X., Yang T. (2020). Postdisaster image-based damage detection and repair cost estimation of reinforced concrete buildings using dual convolutional neural networks. Comput.-Aided Civ. Infrastruct. Eng..

[56] Azimi M., Pekcan G. (2020). Structural Health Monitoring Using Extremely Compressed Data through Deep Learning. Comput.-Aided Civ. Infrastruct. Eng..

[57] Tang Z., Chen Z., Bao Y., Li H. (2019). Convolutional neural network-based data anomaly detection method using multiple information for structural health monitoring. Struct. Control.Health Monit..

[58] Wu R.T., Jahanshahi M.R. (2018). Deep Convolutional Neural Network for Structural Dynamic Response Estimation and System Identification. J. Eng. Mech..

[59] Oh B.K., Glisic B., Kim Y., Park H.S. (2019). Convolutional neural network-based wind-induced response estimation model for tall buildings. Comput.-Aided Civ. Infrastruct. Eng..

[60] Khodabandehlou H., Pekcan G., Fadali M.S. (2019). Vibration-based structural condition assessment using convolution neural networks. Struct. Control. Health Monit..

[61] Li S., Sun L. (2020). Detectability of Bridge-Structural Damage Based on Fiber-Optic Sensing through Deep-Convolutional Neural Networks. J. Bridge Eng..

[62] Pathirage C.S.N., Li J., Li L., Hao H., Liu W., Ni P. (2018). Structural damage identification based on autoencoder neural networks and deep learning. Eng. Struct..

[63] Lu Z.R., Wang L. (2017). An enhanced response sensitivity approach for structural damage identification: Convergence and performance. Int. J. Numer. Methods Eng..

[64] Teng S., Chen G., Liu G., Lv J., Cui F. (2019). Modal Strain Energy-Based Structural Damage Detection Using Convolutional Neural Networks. Appl. Sci..

[65] Sajedi S.O., Liang X. (2019). Vibration-based semantic damage segmentation for large-scale structural health monitoring. Comput.-Aided Civ. Infrastruct. Eng..

[66] Li S., Zuo X., Li Z., Wang H. (2020). Applying Deep Learning to Continuous Bridge Deflection Detected by Fiber Optic Gyroscope for Damage Detection. Sensors.

[67] Lopez-Pacheco M., Morales-Valdez J., Yu W. (2015). Frequency domain CNN and dissipated energy approach for damage detection in building structures. Soft Comput..

[68] Hung D.V., Hung H.M., Anh P.H., Thang N.T. (2020). Structural damage detection using hybrid deep learning algorithm. J. Sci. Technol. Civ. Eng..

[69] Ding Z., Li J., Hao H. (2020). Structural damage identification by sparse deep belief network using uncertain and limited data. Struct. Control. Health Monit..

[70] Fan G., Li J., Hao H. (2020). Vibration Signal Denoising for Structural Health Monitoring by Residual Convolutional Neural Networks. Measurement.

[71] Gatti M. (2019). Structural health monitoring of an operational bridge: A case study. Eng. Struct..

[72] Kim H., Ahn E., Shin M., Sim S.-H. (2019). Crack and noncrack classification from concrete surface images using machine learning. Struct. Health Monit..

[73] Dorafshan S., Maguire M., Hoffer N.V., Coopmans C. (2017). Fatigue Crack Detection Using Unmanned Aerial Systems in Under-Bridge Inspection. Ida. Transp. Dep..

[74] Dorafshan S., Thomas R.J., Maguire M. (2018). Fatigue crack detection using unmanned aerial systems in fracture critical inspection of steel bridges. J. Bridge Eng..

[75] Jahanshahi M.R., Masri S.F. (2012). Adaptive vision-based crack detection using 3D scene reconstruction for condition assessment of structures. Autom. Constr..

[76] Li L., Wang Q., Zhang G., Shi L., Dong J., Jia P. (2018). A method of detecting the cracks of concrete undergo high-temperature. Constr. Build. Mater..

[77] Hamrat M., Boulekbache B., Chemrouk M., Amziane S. (2016). Flexural cracking behavior of normal strength, high strength and high strength fiber concrete beams, using Digital Image Correlation technique. Constr. Build. Mater..

[78] Rimkus A., Podviezko A., Gribniak V. (2015). Processing Digital Images for Crack Localization in Reinforced Concrete Members. Procedia Eng..

[79] Abdel-Qader I., Abudayyeh O., Kelly M.E. (2003). Analysis of edge-detection techniques for crack identification in bridges. J. Comput. Civ. Eng..

[80] Lim R.S., La H.M., Sheng W. (2014). A Robotic Crack Inspection and Mapping System for Bridge Deck Maintenance. IEEE Trans. Autom. Sci. Eng..

[81] Talab A.M.A., Huang Z., Xi F., HaiMing L. (2016). Detection crack in image using Otsu method and multiple filtering in image processing techniques. Optimacal.

[82] Yamaguchi T., Nakamura S., Saegusa R., Hashimoto S. (2008). Image-based crack detection for real concrete surfaces. IEEJ Trans. Electr. Electron. Eng..

[83] Ebrahimkhanlou A., Farhidzadeh A., Salamone S. (2016). Multifractal analysis of crack patterns in reinforced concrete shear walls. Struct. Health Monit..

[84] Spencer B.F., Hoskere V., Narazaki Y. (2019). Advances in Computer Vision-Based Civil Infrastructure Inspection and Monitoring. Engineering.

[85] Feng D., Feng M.Q. (2016). Vision-based multipoint displacement measurement for structural health monitoring. Struct. Control. Health Monit..

[86] Lecompte D., Vantomme J., Sol H. (2006). Crack detection in a concrete beam using two different camera techniques. Struct. Health Monit..

[87] Jahanshahi M.R., Masri S.F. (2012). Parametric performance evaluation of wavelet-based corrosion detection algorithms for condition assessment of civil infrastructure systems. J. Comput. Civ. Eng..

[88] Dawood T., Zhu Z., Zayed T. (2017). Machine vision-based model for spalling detection and quantification in subway networks. Autom. Constr..

[89] German S., Brilakis I., DesRoches R. (2012). Rapid entropy-based detection and properties measurement of concrete spalling with machine vision for post-earthquake safety assessments. Adv. Eng. Inform..

[90] Vaghefi K., Ahlborn T.M., Harris D.K., Brooks C.N. (2013). Combined imaging technologies for concrete bridge deck condition assessment. J. Perform. Constr. Facil..

[91] Mei Q., Gül M., Azim M.R. (2020). Densely connected deep neural network considering connectivity of pixels for automatic crack detection. Autom. Constr..

[92] Xu Y., Bao Y., Chen J., Zuo W., Li H. (2018). Surface fatigue crack identification in steel box girder of bridges by a deep fusion convolutional neural network based on consumer-grade camera images. Struct. Health Monit..

[93] Ali R., Cha Y.J. (2019). Subsurface damage detection of a steel bridge using deep learning and uncooled micro-bolometer. Constr. Build. Mater..

[94] Fu G., Sun P., Zhu W., Yang J., Cao Y., Yang M.Y., Cao Y. (2019). A deep-learning-based approach for fast and robust steel surface defects classification. Opt. Lasers Eng..

[95] Dorafshan S., Thomas R.J., Maguire M. (2018). Comparison of deep convolutional neural networks and edge detectors for image-based crack detection in concrete. Constr. Build. Mater..

[96] Kim H., Ahn E., Cho S., Shin M., Sim S.-H. (2017). Comparative analysis of image binarization methods for crack identification in concrete structures. Cem. Concr. Res..

[97] Liu Y., Cho S., Spencer Jr B.F., Fan J. (2014). Automated assessment of cracks on concrete surfaces using adaptive digital image processing. Smart Struct. Syst..

[98] Giakoumis I., Nikolaidis N., Pitas I. (2006). Digital image processing techniques for the detection and removal of cracks in digitized paintings. IEEE Trans. Image Process..

[99] Jahanshahi M.R., Kelly J.S., Masri S.F., Sukhatme G.S. (2009). A survey and evaluation of promising approaches for automatic image-based defect detection of bridge structures. Struct. Infrastruct. Eng..

[100] Yang X., Li H., Yu Y., Luo X., Huang T., Yang X. (2018). Automatic pixel-level crack detection and measurement using fully convolutional network. Comput.-Aided Civ. Infrastruct. Eng..

[101] Mei Q., Gül M. (2020). Multi-level feature fusion in densely connected deep-learning architecture and depth-first search for crack segmentation on images collected with smartphones. Struct. Health Monit..

[102] Jahanshahi M.R., Masri S.F. (2013). A new methodology for non-contact accurate crack width measurement through photogrammetry for automated structural safety evaluation. Smart Mater. Struct..

[103] Yeum C.M., Dyke S.J. (2015). Vision-based automated crack detection for bridge inspection. Comput.-Aided Civ. Infrastruct. Eng..

[104] Comparative Study of Deep Learning Software Frameworks. https://arxiv.org/abs/1511.06435.

[105] Mohan A., Poobal S. (2018). Crack detection using image processing: A critical review and analysis. Alex. Eng. J..

[106] Cha Y.J., Choi W., Büyüköztürk O. (2017). Deep Learning-Based Crack Damage Detection Using Convolutional Neural Networks. Comput.-Aided Civ.Infrastruct. Eng..

[107] Otsu N. (1979). A threshold selection method from gray-level histograms. IEEE Trans. Syst. Man Cybern..

[108] Rabah M., Elhattab A., Fayad A. (2013). Automatic concrete cracks detection and mapping of terrestrial laser scan data. NRIAG J. Astron. Geophys..

[109] Prasanna P., Dana K.J., Gucunski N., Basily B.B., La H.M., Lim R.S., Parvardeh H. (2016). Automated crack detection on concrete bridges. IEEE Trans. on Autom. Sci. Eng..

[110] Rawat W., Wang Z. (2017). Deep Convolutional Neural Networks for Image Classification: A Comprehensive Review. Neural Comput..

[111] Badrinarayanan V., Kendall A., Cipolla R. (2017). Segnet: A deep convolutional encoder-decoder architecture for image segmentation. IEEE Trans. Pattern Anal. Mach. Intell..

[112] Long J., Shelhamer E., Darrell T. Fully convolutional networks for semantic segmentation. Proceedings of the IEEE conference on computer vision and pattern recognition.

[113] Chen F., Jahanshahi M.R. (2018). NB-CNN: Deep Learning-Based Crack Detection Using Convolutional Neural Network and Naïve Bayes Data Fusion. IEEE Trans. Ind. Electron..

[114] Li G., Ma B., He S., Ren X., Liu Q. (2020). Automatic Tunnel Crack Detection Based on U-Net and a Convolutional Neural Network with Alternately Updated Clique. Sensors.

[115] Soloviev A., Sobol B., Vasiliev P. (2019). Identification of Defects in Pavement Images Using Deep Convolutional Neural Networks. Adv. Mater..

[116] Li B., Wang K.C., Zhang A., Yang E., Wang G. (2020). Automatic classification of pavement crack using deep convolutional neural network. Int. J. Pavement Eng..

[117] Tong Z., Gao J., Han Z., Wang Z. (2018). Recognition of asphalt pavement crack length using deep convolutional neural networks. Road Mater. Pavement Des..

[118] Fan Z., Li C., Chen Y., Mascio P.D., Chen X., Zhu G., Loprencipe G. (2020). Ensemble of Deep Convolutional Neural Networks for Automatic Pavement Crack Detection and Measurement. Coatings.

[119] Automatic Pavement Crack Detection Based on Structured Prediction with the Convolutional Neural Network. https://arxiv.org/abs/1802.02208.

[120] Wang K.C.P., Zhang A., Li J.Q., Fei Y., Chen C., Li B. (2017). Deep Learning for Asphalt Pavement Cracking Recognition Using Convolutional Neural Network. Airfield Highw. Pavements.

[121] FPCNet: Fast Pavement Crack Detection Network Based on Encoder-Decoder Architecture. https://arxiv.org/abs/1907.02248.

[122] Kim B., Cho S. (2018). Automated Vision-Based Detection of Cracks on Concrete Surfaces Using a Deep Learning Technique. Sensors.

[123] Cha Y.J., Choi W. (2017). Vision-Based Concrete Crack Detection Using a Convolutional Neural Network. Proc. Dyn. Civ. Struct..

[124] An Y.K., Jang K.Y., Kim B., Cho S. Deep learning-based concrete crack detection using hybrid images. Proceedings of the Sensors and Smart Structures Technologies for Civil, Mechanical, and Aerospace Systems.

[125] Jang K., An Y.K., Kim B., Cho S. (2020). Automated crack evaluation of a high-rise bridge pier using a ring-type climbing robot. Comput.-Aided Civ. Infrastruct. Eng..

[126] Ali R., Gopal D.L., Cha Y.-J. Vision-based concrete crack detection technique using cascade features. Proceedings of the Sensors and Smart Structures Technologies for Civil, Mechanical, and Aerospace Systems.

[127] Ramana L., Choi W., Cha Y.-J. (2019). Fully automated vision-based loosened bolt detection using the Viola–Jones algorithm. Struct. Health Monit..

[128] Ramana L., Choi W., Cha Y.-J. (2017). Automated vision-based loosened bolt detection using the cascade detector. Sens. Instrum..

[129] Cha Y.J., You K., Choi W. (2016). Vision-based detection of loosened bolts using the Hough transform and support vector machines. Autom. Constr..

[130] Yeum C.M., Dyke S.J., Ramirez J. (2018). Visual data classification in post-event building reconnaissance. Eng. Struct..

[131] Xu Y., Wei S., Bao Y., Li H. (2019). Automatic seismic damage identification of reinforced concrete columns from images by a region-based deep convolutional neural network. Struct. Control. Health Monit..

[132] Girshick R. Fast R-CNN. Proceedings of the IEEE International Conference on Computer Vision.

[133] Ren S., He K., Girshick R., Sun J. (2015). Faster R-CNN: Towards real-time object detection with region proposal networks. Proc. Adv. Neural Inf. Process. Syst..

[134] Wang N., Zhao X., Zou Z., Zhao P., Qi F. (2015). Autonomous damage segmentation and measurement of glazed tiles in historic buildings via deep learning. Comput.-Aided Civ. Infrastruct. Eng..

[135] Deng L., Chu H.-H., Shi P., Wang W., Kong X. (2020). Region-Based CNN Method with Deformable Modules for Visually Classifying Concrete Cracks. Appl. Sci..

[136] Zhang X., Rajan D., Story B. (2015). Concrete crack detection using context-aware deep semantic segmentation network. Comput.-Aided Civ. Infrastruct. Eng..

[137] Zhang A., Wang K.C., Li B., Yang E., Dai X., Peng Y., Fei Y., Liu Y., Li J.Q., Chen C. (2017). Automated pixel-level pavement crack detection on 3D asphalt surfaces using a deep-learning network. Comput.-Aided Civ. Infrastruct. Eng..

[138] Xu Y., Li S., Zhang D., Jin Y., Zhang F., Li N., Li H. (2018). Identification framework for cracks on a steel structure surface by a restricted Boltzmann machines algorithm based on consumer-grade camera images. Struct. Control. Health Monit..

[139] Hoskere V., Narazaki Y., Hoang T.A., Spencer B.F. Vision-based Structural Inspection using Multiscale Deep Convolutional Neural Networks. Proceedings of the 3rd Huixian International Forum on Earthquake Engineering for Young Researchers.

[140] Hoskere V., Narazaki Y., Hoang T.A., Spencer B.F. Towards Automated Post-Earthquake Inspections with Deep Learning-based Condition-Aware Models. Proceedings of the 7th World Conference on Structural Control and Monitoring, 7WCSCM.

[141] Yeum C.M., Choi J., Dyke S.J. (2018). Automated region-of-interest localization and classification for vision-based visual assessment of civil infrastructure. Struct. Health Monit..

[142] Gao Y., Mosalam K.M. (2018). Deep Transfer Learning for Image-Based Structural Damage Recognition. Comput.-Aided Civ. Infrastruct. Eng..

[143] Liang X. (2018). Image-based post-disaster inspection of reinforced concrete bridge systems using deep learning with Bayesian optimization. Comput.-Aided Civ. Infrastruct. Eng..

[144] Narazakia Y., Hoskerea V., Hoanga T.A., Spencer B.F. Automated Bridge Component Recognition using Video Data. Proceedings of the 7th World Conference on Structural Control and Monitoring, 7WCSCM.

[145] Alcantarilla P.F., Stent S., Ros G., Arroyo R., Gherardi R. (2018). Street-view change detection with deconvolutional networks. Auton. Robot..

[146] Stent S., Gherardi R., Stenger B., Soga K., Cipolla R. (2016). Visual change detection on tunnel linings. Mach. Vis. Appl..

[147] Dorafshan S., Maguire M. (2018). Bridge inspection: Human performance, unmanned aerial systems and automation. J. Civ. Struct. Health Monit..

[148] Dorafshan S., Thomas R.J., Maguire M. (2019). Benchmarking Image Processing Algorithms for Unmanned Aerial System-Assisted Crack Detection in Concrete Structures. Infrastructure.

[149] Zink J., Lovelace B. (2015). Unmanned Aer. Veh. Bridge Insp. Demonstr. Proj..

[150] Wells J., Lovelace B., Engineers C. (2017). Unmanned Aircr. Syst. Bridge Insp. Demonstr. Proj. Phase II Final. Rep..

[151] Tomiczek A.P., Bridge J.A., Ifju P.G., Whitley T.J., Tripp C.S., Ortega A.E., Poelstra J.J., Gonzalez S.A. Small Unmanned Aerial Vehicle (sUAV) Inspections in GPS Denied Area beneath Bridges. Proceedings of the Structures Congress.

[152] Duque L., Seo J., Wacker J. (2018). Synthesis of unmanned aerial vehicle applications for infrastructures. J. Perform. Constr. Facil..

[153] Kim H., Lee J., Ahn E., Cho S., Shin M., Sim S.-H. (2017). Concrete crack identification using a UAV incorporating hybrid image processing. Sensors.

[154] Morgenthal G., Hallermann N. (2014). Quality assessment of unmanned aerial vehicle (UAV) based visual inspection of structures. Adv. Struct. Eng..

[155] Yoon H., Shin J., Spencer Jr B.F. (2018). Structural displacement measurement using an unmanned aerial system. Comput.-Aided Civ. Infrastruct. Eng..

[156] Pereira F.C., Pereira C.E. (2015). Embedded image processing systems for automatic recognition of cracks using UAVs. IFAC-PapersOnLine.

[157] Jang K., Kim N., An Y.-K. (2019). Deep learning–based autonomous concrete crack evaluation through hybrid image scanning. Struct. Health Monit..

[158] Kim I.-H., Jeon H., Baek S.-C., Hong W.-H., Jung H.-J. (2018). Application of crack identification techniques for an aging concrete bridge inspection using an unmanned aerial vehicle. Sensors.

[159] Kang D., Cha Y.-J. Damage detection with an autonomous UAV using deep learning. Proceedings of the Sensors and Smart Structures Technologies for Civil, Mechanical, and Aerospace Systems.

[160] Huynh T.-C., Park J.-H., Jung H.-J., Kim J.-T. (2019). Quasi-autonomous bolt-loosening detection method using vision-based deep learning and image processing. Autom. Constr..

[161] Chaiyasarn K., Khan W., Ali L., Sharma M., Brackenbury D., DeJong M. Crack Detection in Masonry Structures using Convolutional Neural Networks and Support Vector Machines. Proceedings of the International Symposium on Automation and Robotics in Construction.

[162] Lei B., Wang N., Xu P., Song G. (2018). New crack detection method for bridge inspection using UAV incorporating image processing. J. Aerosp. Eng..

[163] Duarte D., Nex F., Kerle N., Vosselman G. (2018). Multi-resolution feature fusion for image classification of building damages with convolutional neural networks. Remote. Sens..

[164] Hoskere V., Park J.-W., Yoon H., Spencer Jr B.F. (2019). Vision-Based Modal Survey of Civil Infrastructure Using Unmanned Aerial Vehicles. J. Struct. Eng..

[165] Gopalakrishnan K. (2018). Symposium on Automation and Robotics. Deep Learn. Pavement Image Anal. Autom. Distress Detect. Rev..

[166] Zhang Y., Sun X., Loh K.J., Su W., Xue Z., Zhao X. (2019). Autonomous bolt loosening detection using deep learning. Struct. Health Monit..

[167] Beckman G.H., Polyzois D., Cha Y.J. (2019). Deep learning-based automatic volumetric damage quantification using depth camera. Autom. Constr..

[168] Zhao X., Han R., Yu Y., Hu W., Jiao D., Mao X., Li M., Ou J. (2016). Smartphone-based mobile testing technique for quick bridge cable–force measurement. J. Bridge Eng..

[169] Li S., Zhao X. Convolutional neural networks-based crack detection for real concrete surface. Proceedings of the Sensors and Smart Structures Technologies for Civil, Mechanical, and Aerospace Systems.

[170] Li S., Zhao X. (2019). Image-Based Concrete Crack Detection Using Convolutional Neural Network and Exhaustive Search Technique. Adv. Civ. Eng..

[171] Wang N., Zhao X., Zhao P., Zhang Y., Zou Z., Ou J. (2019). Automatic damage detection of historic masonry buildings based on mobile deep learning. Autom. Constr..

[172] Pauly L., Hogg D., Fuentes R., Peel H. Deeper networks for pavement crack detection. Proceedings of the 34th ISARC.

[173] Maeda H., Sekimoto Y., Seto T., Kashiyama T., Omata H. (2018). Road damage detection and classification using deep neural networks with smartphone images. Comput.-Aided Civ. Infrastruct. Eng..

[174] Pan S.J., Yang Q. (2010). A survey on transfer learning. IEEE Trans. Knowl. Data Eng..

[175] Zhang K., Cheng H.D., Zhang B. (2018). Unified Approach to Pavement Crack and Sealed Crack Detection Using Preclassification Based on Transfer Learning. J. Comput. Civ. Eng..

[176] Gopalakrishnan K., Khaitan S.K., Choudhary A., Agrawal A. (2017). Deep Convolutional Neural Networks with transfer learning for computer vision-based data-driven pavement distress detection. Constr. Build. Mater..

[177] Perez H., Tah J.H., Mosavi A. (2019). Deep Learning for Detecting Building Defects Using Convolutional Neural Networks. Sensors.

[178] Özgenel Ç.F., Sorguç A.G. Performance Comparison of Pretrained Convolutional Neural Networks on Crack Detection in Buildings. Proceedings of the International Symposium on Automation and Robotics in Construction.

[179] Wu R.T., Singla A., Jahanshahi M.R., Bertino E., Ko B.J., Verma D. (2019). Pruning deep convolutional neural networks for efficient edge computing in condition assessment of infrastructures. Comput.-Aided Civ. Infrastruct. Eng..

[180] Deng J., Dong W., Socher R., Li L.J., Li K., Fei-Fei L. ImageNet: A large-scale hierarchical image database. Proceedings of the IEEE Conference on Computer Vision and Pattern Recognition.

[181] Szegedy C., Vanhoucke V., Ioffe S., Shlens J., Wojna Z. Rethinking the inception architecture for computer vision. Proceedings of the IEEE conference on computer vision and pattern recognition.

[182] He K., Zhang X., Ren S., Sun J. Deep residual learning for image recognition. Proceedings of the IEEE conference on computer vision and pattern recognition.

[183] Serre T., Wolf L., Bileschi S., Riesenhuber M., Poggio T. (2007). Robust object recognition with cortex-like mechanisms. IEEE Trans. Pattern Anal. Mach. Intell..

[184] Zeiler M.D., Fergus R. Visualizing and understanding convolutional networks. Proceedings of the European Conference on Computer Vision.

[185] Zhang A., Wang K.C., Fei Y., Liu Y., Tao S., Chen C., Li J.Q., Li B. (2018). Deep learning–based fully automated pavement crack detection on 3D asphalt surfaces with an improved CrackNet. J. Comput. Civ. Eng..

[186] Artificial Intelligence Assisted Infrastructure Assessment Using Mixed Reality Systems. https://arxiv.org/abs/1812.05659.

[187] Gopalakrishnan K., Gholami H., Vidyadharan A., Choudhary A., Agrawal A. (2018). Crack Damage Detection in Unmanned Aerial Vehicle Images of Civil Infrastructure Using Pre-Trained Deep Learning Model. Int. J. Traffic Transp. Eng.

[188] Park S., Bang S., Kim H., Kim H. (2019). Patch-Based Crack Detection in Black Box Images Using Convolutional Neural Networks. J. Comput. Civ. Eng..

[189] Dung C.V., Sekiya H., Hirano S., Okatani T., Miki C. (2019). A vision-based method for crack detection in gusset plate welded joints of steel bridges using deep convolutional neural networks. Autom. Constr..

[190] Narazaki Y., Hoskere V., Hoang T.A., Spencer B.J. (2019). Vision-based automated bridge component recognition with high-level scene consistency. Comput.-Aided Civ. Infrastruct. Eng..

[191] Narazaki Y., Hoskere V., Hoang T.A., Spencer B.F. Automated Vision-Based Bridge Component Extraction Using Multiscale Convolutional Neural Networks. https://arxiv.org/abs/1812.05659.

[192] Liu H., Zhang Y. (2019). Image-driven structural steel damage condition assessment method using deep learning algorithm. Measurement.

[193] Nahata D., Mulchandani H., Bansal S., Muthukumar G. (2019). Post-Earthquake. Assessment. Buildlings Using Deep Learning. J. Comput. Civ. Eng..

[194] Bang S., Park S., Kim H., Kim H. (2019). Encoder–decoder network for pixel-level road crack detection in black-box images. Comput.-Aided Civ. Infrastruct. Eng..

[195] Szegedy C., Ioffe S., Vanhoucke V., Alemi A.A. Inception-v4, inception-resnet and the impact of residual connections on learning. Proceedings of the 31st AAAI Conference on Artificial Intelligence.

[196] Wang N., Zhao Q., Li S., Zhao X., Zhao P. (2018). Damage classification for masonry historic structures using convolutional neural networks based on still images. Comput.-Aided Civ. Infrastruct. Eng..

[197] Dick K., Russell L., Souley Dosso Y., Kwamena F., Green J.R. (2019). Deep learning for critical infrastructure resilience. J. Infrastruct. Syst..

[198] Ni F., Zhang J., Chen Z. (2018). Zernike-moment measurement of thin-crack width in images enabled by dual-scale deep learning. Comput.-Aided Civ. and Infrastruct. Eng..

[199] Narazaki Y., Hoskere V., Hoang T.A., Spencer B.F. Vision-based Automated Bridge Component Recognition Integrated With High-level Scene Understanding. Proceedings of the 13th International Workshop on Advanced Smart Materials and Smart Structures Technology.

[200] Ahmed H., La H., Pekcan G. Rebar Detection and Localization for Non-Destructive Infrastructure Evaluation of Bridges using Deep Residual Networks. Proceedings of the 14th International Symposium on Visual Computing: ISVC’19.

[201] Kim B., Lee Y., Cho S. Deep learning-based rapid inspection of concrete structures. Proceedings of the Sensors and Smart Structures Technologies for Civil, Mechanical, and Aerospace Systems.

[202] Zhao X., Li S., Su H., Zhou L., Loh K.J. Image-Based Comprehensive Maintenance and Inspection Method for Bridges Using Deep Learning. Proceedings of the ASME Conference on Smart Materials, Adaptive Structures and Intelligent Systems.

[203] Dorafshan S., Thomas R.J., Coopmans C., Maguire M. Deep learning neural networks for suas-assisted structural inspections: Feasibility and application. Proceedings of the 2018 International Conference on Unmanned Aircraft Systems (ICUAS).

[204] Suh G., Cha Y.-J. Deep faster R-CNN-based automated detection and localization of multiple types of damage. Proceedings of the Sensors and Smart Structures Technologies for Civil, Mechanical, and Aerospace Systems 2018.

[205] Beckman G.H., Polyzois D., Cha Y.-J. Automated volumetric damage detection and quantification using region-based convolution neural networks and an inexpensive depth camera. Proceedings of the Sensors and Smart Structures Technologies for Civil, Mechanical, and Aerospace Systems.

[206] SDNET2018: A Concrete Crack Image Dataset for Machine Learning Applications. https://digitalcommons.usu.edu/all_datasets/48/.

[207] Gao Y., Kong B., Mosalam K.M. (2019). Deep leaf-bootstrapping generative adversarial network for structural image data augmentation. Comput.-Aided Civ. Infrastruct. Eng..

[208] Zhang Y., Miyamori Y., Mikami S., Saito T. (2019). Vibration-based structural state identification by a 1-dimensional convolutional neural network. Comput.-Aided Civ. Infrastruct. Eng..

[209] Yu Y., Wang C., Gu X., Li J. (2019). A novel deep learning-based method for damage identification of smart building structures. Struct. Health Monit..

[210] Gulgec N.S., Takáč M., Pakzad S.N. (2019). Convolutional Neural Network Approach for Robust Structural Damage Detection and Localization. J. Comput. Civ. Eng..

[211] Ye X.W., Jin T., Chen P.Y. (2019). Structural crack detection using deep learning–based fully convolutional networks. Adv. Struct. Eng..

[212] Gao Y., Li K., Mosalam K., Günay S. Deep Residual Network with Transfer Learning for Imagebased Structural Damage Recognition. Proceedings of the Eleventh US National Conference on Earthquake Engineering, Integrating Science, Engineering & Policy.

[213] Silva W.R.L.d., Lucena D.S.d. (2001). Concrete cracks detection based on deep learning image classification. Proc. Multidiscip. Digit. Publ. Inst. Proc..

[214] Sharma M., Anotaipaiboon W., Chaiyasarn K. Crack Detection in Masonry Structures using Convolutional Neural Networks and Support Vector Machines. Proceedings of the 35th International Symposium on Automation and Robotics in Construction (ISARC 2018).

[215] Kumar S.S., Abraham D.M., Jahanshahi M.R., Iseley T., Starr J. (2018). Automated defect classification in sewer closed circuit television inspections using deep convolutional neural networks. Autom. Constr..

[216] De Oliveira M., Monteiro A., Vieira Filho J. (2018). A New Structural Health Monitoring Strategy Based on PZT Sensors and Convolutional Neural Network. Sensors.

[217] Li Y., Zhao W., Zhang X., Zhou Q. (2018). A two-stage crack detection method for concrete bridges using Convolutional Neural Networks. IEICE Trans. Inf. Syst..

[218] Ji M., Liu L., Buchroithner M. (2018). Identifying Collapsed Buildings Using Post-Earthquake Satellite Imagery and Convolutional Neural Networks: A Case Study of the 2010 Haiti Earthquake. Remote. Sens..

[219] Modarres C., Astorga N., Droguett E.L., Meruane V. (2018). Convolutional neural networks for automated damage recognition and damage type identification. Struct. Control. Health Monit..

[220] Pouyanfar S., Sadiq S., Yan Y., Tian H., Tao Y., Reyes M.P., Shyu M.-L., Chen S.C., Iyengar S. (2018). A survey on deep learning: Algorithms, techniques, and applications. ACM Comput. Surv. (CSUR).

[221] Abadi M., Barham P., Chen J., Chen Z., Davis A., Dean J., Devin M., Ghemawat S., Irving G., Isard M. Tensorflow: A system for large-scale machine learning. Proceedings of the OSDI.

[222] Fonnegra R.D., Blair B., Díaz G.M. Performance comparison of deep learning frameworks in image classification problems using convolutional and recurrent networks. Proceedings of the 2017 IEEE Colombian Conference on Communications and Computing (COLCOM).

[223] Shi S., Wang Q., Xu P., Chu X. Benchmarking state-of-the-art deep learning software tools. Proceedings of the 2016 7th International Conference on Cloud Computing and Big Data (CCBD).

[224] Chollet F. Keras. https://www.scirp.org/(S(351jmbntvnsjt1aadkposzje))/reference/ReferencesPapers.aspx?ReferenceID=1887532.

[225] Jia Y., Shelhamer E., Donahue J., Karayev S., Long J., Girshick R., Guadarrama S., Darrell T. Caffe: Convolutional architecture for fast feature embedding. Proceedings of the 22nd ACM international conference on Multimedia.

[226] Zhang T., Biswal S., Wang Y. (2019). SHMnet: Condition Assessment of Bolted Connection with Beyond Human-level Performance. Struct. Health Monit..

[227] Roohi M., Hernandez E.M., Rosowsky D. (2019). Nonlinear Seismic Response Reconstruction and Performance Assessment of Instrumented Wood-frame Buildings—Validation using NEESWood Capstone Full-Scale Tests. Struct. Control. Health Monit..

[228] Aono K., Kondapalli S.H., Lajnef N., Pekcan G., Faridazar F., Chakrabartty S. Self-powered Sensors to Facilitate Infrastructural Internet-of-Things for Smart Structures. Proceedings of the 13th International Workshop on Advanced Smart Materials and Smart Structures Technology.

[229] Fu Y., Mechitov K., Hoang T., Kim J.R., Lee D.H., Spencer B.F. (2019). Development and full-scale validation of high-fidelity data acquisition on a next-generation wireless smart sensor platform. Adv. Struct. Eng..

[230] Yu J., Meng X., Yan B., Xu B., Fan Q., Xie Y. (2020). Global Navigation Satellite System-based positioning technology for structural health monitoring: A review. Struct. Control Health Monit..

[231] Design of Structural Vibration Control Using Smart Materials and Devices for Earthquake-Resistant and Resilient Buildings. https://library.ndsu.edu/ir/handle/10365/28588.

[232] Azimi M., Yeznabad A.M. (2020). Swarm-Based Parallel Control of Adjacent Irregular Buildings Considering Soil–Structure Interaction. J. Sens. Actuator Netw..

[233] Mnih V., Kavukcuoglu K., Silver D., Rusu A.A., Veness J., Bellemare M.G., Graves A., Riedmiller M., Fidjeland A.K., Ostrovski G. (2015). Human-level control through deep reinforcement learning. Nature.

[234] Zhu M., McKenna F., Scott M.H. (2018). OpenSeesPy: Python library for the OpenSees finite element framework. Software.

[235] Brockman G., Cheung V., Pettersson L., Schneider J., Schulman J., Tang J., Zaremba W. Openai Gym. https://arxiv.org/abs/1606.01540.

